# Targeting Reactive Species Stress and Nutrient Sensing to Enhance NK-Cell Function: Mechanistic Strategies for Overcoming Pancreatic Cancer Progression and Resistance Through Supplement Therapy

**DOI:** 10.3390/ijms27177893

**Published:** 2026-09-04

**Authors:** Sara Fanijavadi, Lars Henrik Jensen

**Affiliations:** 1 SF-Medic Oncology Hub, 28924 Madrid, Spain; 2 Cancer Polyclinic, Levanger Hospital, 7601 Levanger, Norway; 3 Department of Oncology, Vejle Hospital, University Hospital of Southern Denmark, 7100 Vejle, Denmark; lars.henrik.jensen@rsyd.dk; 4 Department of Oncology, Institute of Regional Health Research, University of Southern Denmark, 7100 Vejle, Denmark

**Keywords:** pancreatic cancer, NK cell, reactive species stress, nutrient sensing, antioxidant, micronutrient, triple NK-cell approach, treatment resistance

## Abstract

Pancreatic ductal adenocarcinoma (PDAC) remains one of the deadliest malignancies worldwide because of delayed diagnosis, rapid metastatic progression, profound immune suppression, and limited therapeutic responsiveness. The pancreatic tumor microenvironment is characterized by severe hypoxia, stromal desmoplasia, metabolic stress, and oxidative imbalance, all of which contribute to tumor progression and resistance to therapy. Among immune cells involved in antitumor defense, natural killer (NK) cells play an important role through direct cytotoxicity and cytokine production. However, NK-cell activity is frequently impaired in PDAC due to oxidative stress, altered nutrient availability, mitochondrial dysfunction, and dysregulated signaling pathways such as AMP-activated protein kinase (AMPK) and mammalian target of rapamycin (mTOR). This review examines current evidence regarding the interactions among redox biology, nutrient sensing, NK-cell metabolism, and pancreatic cancer progression. Importantly, much of the available evidence derives from in vitro studies, animal models, or early-phase clinical investigations, and several findings remain controversial or inconsistent. Further well-designed clinical trials are needed to determine whether nutritional interventions, vitamin supplementation, and strategies targeting metabolic and redox pathways can safely and effectively enhance NK-cell function and improve clinical outcomes in patients with PDAC.

## 1. Introduction

Pancreatic ductal adenocarcinoma (PDAC) remains one of the most aggressive and treatment-resistant malignancies, with a persistently poor prognosis and high mortality rate despite advances in surgery, chemotherapy, radiotherapy, and targeted therapies [1]. Most patients are diagnosed at advanced stages because early disease is frequently asymptomatic, and even patients who undergo surgical resection commonly develop recurrence or metastatic progression [2]. Systemic chemotherapy remains the standard treatment for advanced PDAC; however, therapeutic responses are often short-lived due to intrinsic and acquired resistance mechanisms [3]. Consequently, there is an urgent need to better understand the biological factors underlying PDAC progression, immune evasion, and treatment resistance.

The resistance of PDAC to conventional therapies is strongly influenced by the tumor microenvironment (TME), which is characterized by dense desmoplasia, hypoxia, metabolic stress, inflammatory signaling, and profound immune suppression [4]. Within this hostile microenvironment, cancer cells undergo metabolic and redox adaptations that support tumor survival, metastatic potential, and resistance to apoptosis. These alterations also impair antitumor immune responses, particularly the activity of natural killer (NK) cells, which are critical mediators of innate immune surveillance.

One important contributor to PDAC progression and immune dysfunction is redox imbalance involving reactive oxygen species (ROS), reactive nitrogen species (RNS), and reactive sulfur species (RSS) [5]. Under physiological conditions, these reactive species participate in cellular signaling and immune regulation. However, persistent elevation of ROS, RNS, and RSS in the PDAC TME can disrupt redox homeostasis and promote oxidative and nitrosative damage to DNA, proteins, and lipids [6,7,8]. ROS are generated primarily through mitochondrial oxidative phosphorylation, NADPH oxidase activity, inflammatory signaling, and metabolic stress [6]. Elevated ROS levels have been implicated in epithelial–mesenchymal transition (EMT), genomic instability, angiogenesis, inflammatory cytokine production, immune suppression, and chemoresistance in PDAC [6]. Similarly, RNS such as nitric oxide (NO) can modify proteins and signaling molecules through nitrosative reactions, thereby influencing angiogenesis, tumor progression, and immune regulation [7]. RSS, particularly hydrogen sulfide (H_2_S), also contribute to redox signaling and metabolic adaptation within the TME, although their effects appear highly concentration- and context-dependent [8].

Accumulating evidence suggests that persistent redox stress within the PDAC TME contributes to impaired NK-cell activity [9]. Excessive reactive species can reduce NK-cell proliferation, cytokine secretion, tumor infiltration, and cytotoxicity, thereby weakening immune-mediated tumor control [10]. Conversely, modulation of redox balance through selected antioxidants or metabolic interventions has shown the potential to partially restore NK-cell function in preclinical models [9,10]. Nevertheless, evidence supporting these approaches in human PDAC remains limited and sometimes contradictory, emphasizing the need for cautious interpretation.

In parallel with redox dysregulation, altered nutrient sensing pathways play a major role in PDAC progression and immune escape. Cancer cells within the nutrient-deprived TME adapt through metabolic reprogramming involving pathways such as the mechanistic target of rapamycin (mTOR) and AMP-activated protein kinase (AMPK) [11]. mTOR signaling promotes anabolic metabolism, cell growth, and survival, whereas AMPK is activated during energy stress and regulates catabolic pathways to maintain cellular homeostasis [11,12,13]. Dysregulation of these pathways supports tumor adaptation to hypoxia, oxidative stress, and nutrient deprivation while simultaneously affecting immune-cell metabolism and function. In PDAC, persistent activation of PI3K/AKT/mTOR signaling has been associated with tumor progression and resistance to therapy [12]. Although pharmacologic inhibition of mTOR has shown limited clinical benefit as monotherapy, emerging preclinical studies suggest that combining metabolic modulation with immunologic or nutritional approaches may enhance antitumor responses [12].

NK cells are increasingly recognized as important mediators of antitumor immunity in PDAC. Unlike T cells, NK cells can recognize and eliminate stressed or transformed cells independently of major histocompatibility complex (MHC) presentation [14,15]. However, NK-cell function in PDAC is profoundly impaired by the immunosuppressive and metabolically hostile TME. Factors including transforming growth factor-β (TGF-β), hypoxia, oxidative stress, lactate accumulation, and nutrient deprivation collectively suppress NK-cell activation, proliferation, cytokine production, and cytotoxic granule release [15,16,17]. Mitochondrial dysfunction and metabolic exhaustion further compromise NK-cell persistence and tumor infiltration within PDAC lesions [18].

Recent studies have therefore explored whether modulation of redox pathways and nutrient sensing could improve NK-cell function and enhance antitumor immunity in PDAC [19]. Micronutrients, vitamins, minerals, and dietary compounds have attracted attention because some experimental models suggest they may influence oxidative stress responses, mitochondrial metabolism, inflammatory signaling, or NK-cell activity [9,20]. However, the current evidence remains heterogeneous, with many findings derived from in vitro systems, animal models, or observational studies rather than randomized clinical trials in PDAC patients. Furthermore, some micronutrients may exert context-dependent or even opposing effects depending on dosage, timing, tumor stage, and concurrent therapies.

**Figure 1:**
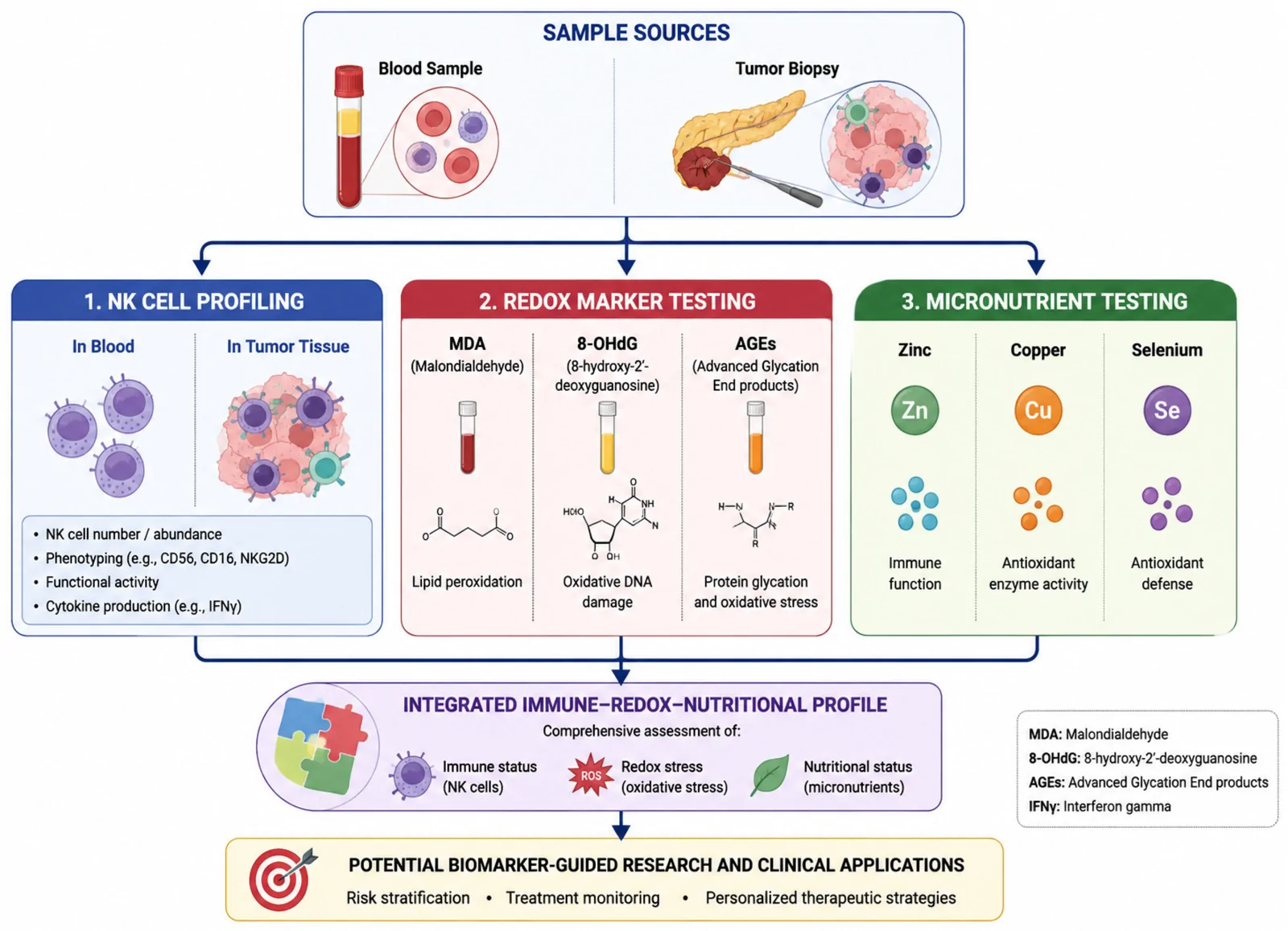
Integrated profiling of NK Cells, redox status, and micronutrients in blood and tumor tissue. Schematic overview of a proposed integrated assessment using blood samples and tumor biopsies from patients with pancreatic ductal adenocarcinoma (PDAC). NK-cell profiling assesses NK-cell abundance, phenotype, and functional characteristics in blood and, where feasible, tumor tissue. Redox marker testing assesses indirect indicators of oxidative stress, including malondialdehyde (MDA), 8-hydroxy-2′-deoxyguanosine (8-OHdG), and advanced glycation end products (AGEs). Micronutrient testing assesses selected nutrients relevant to immune and antioxidant function, including zinc, copper, and selenium. Integration of these three domains may provide a broader assessment of immune status, redox stress, and nutritional status and may support biomarker-guided research and treatment monitoring. This framework is proposed as a research and biomarker-stratification strategy and should not be interpreted as a currently validated clinical diagnostic algorithm.

Therefore, this review aims to critically examine the relationship between redox biology, nutrient sensing, and NK-cell dysfunction in PDAC. We discuss how ROS, RNS, RSS, AMPK, and mTOR pathways influence NK-cell metabolism, cytotoxicity, and tumor infiltration within the pancreatic TME. We also evaluate current evidence regarding micronutrient and antioxidant-based interventions, with emphasis on mechanistic insights, therapeutic limitations, and translational challenges in PDAC management. A comprehensive approach integrating NK-cell profiling, redox biomarkers, and micronutrient assessment in blood and tumor tissue may provide a more complete understanding of immune dysfunction and metabolic stress in PDAC (Figure 1). NK-cell profiling can assess circulating NK-cell abundance and functional status and, where feasible, intratumoral NK-cell status. Redox assessment using indirect markers such as malondialdehyde (MDA), 8-hydroxy-2′-deoxyguanosine (8-OHdG), and advanced glycation end products (AGEs) may help characterize oxidative stress, while micronutrient assessment may identify deficiencies or alterations in nutrients relevant to immune and antioxidant function. Because redox imbalance, nutrient availability, and NK-cell dysfunction are biologically interconnected in the PDAC microenvironment, assessing these parameters together may help identify relationships between nutritional status, oxidative stress, NK-cell function, and treatment response. This framework is proposed as a research and biomarker-stratification strategy and should not be interpreted as a currently validated clinical diagnostic algorithm [21,22].

## 2. NK Cells Within the Innate–Adaptive Immune Network of PDAC

Although this review focuses on NK cells, NK cells are not the only immune cells involved in PDAC progression and immune regulation. The PDAC TME contains both innate and adaptive immune populations that interact with each other and collectively influence tumor growth, immune suppression, and treatment response. In particular, CD4+ T cells, regulatory T cells (Tregs), and cytotoxic T lymphocytes (CTLs) contribute to adaptive immune responses and can influence NK-cell activity within the TME. These interactions highlight that NK-cell dysfunction should be considered within the broader immune network rather than as an isolated process [23,24].

Other innate immune cells, particularly macrophages and neutrophils, also contribute to the immunosuppressive and metabolically stressed PDAC TME. Together with tumor cells and other stromal components, these cells can influence inflammatory signaling, oxidative stress, nutrient availability, and metabolic conditions that affect immune-cell function [25]. Such interactions may further contribute to the hostile environment in which NK cells become metabolically exhausted and functionally impaired.

The focus on NK cells in this review is not intended to imply that NK cells are more important than T cells, neutrophils, macrophages, or other immune populations within the PDAC tumor microenvironment. Rather, NK cells were selected as the principal immune-cell focus because their antitumor activity is closely dependent on metabolic fitness, mitochondrial function, redox homeostasis, and nutrient availability [11,20,26,27,28]. NK-cell cytotoxicity, cytokine production, proliferation, and tumor infiltration are sensitive to metabolic and redox disturbances within the PDAC microenvironment [14,29]. This makes NK cells a useful cellular framework for examining the interaction between nutrient stress, reactive species, metabolic signaling, and immune dysfunction. T cells and neutrophils are also critically involved in PDAC immune regulation [30,31,32,33], but their biology encompasses additional mechanisms that are beyond the specific scope of this review. Thus, NK cells are used here as a focused model rather than as a uniquely superior or more clinically accessible immune-cell population.

## 3. Redox and Metabolic Regulation of NK Cells in PDAC

### 3.1. Redox Mediators

**Figure 2:**
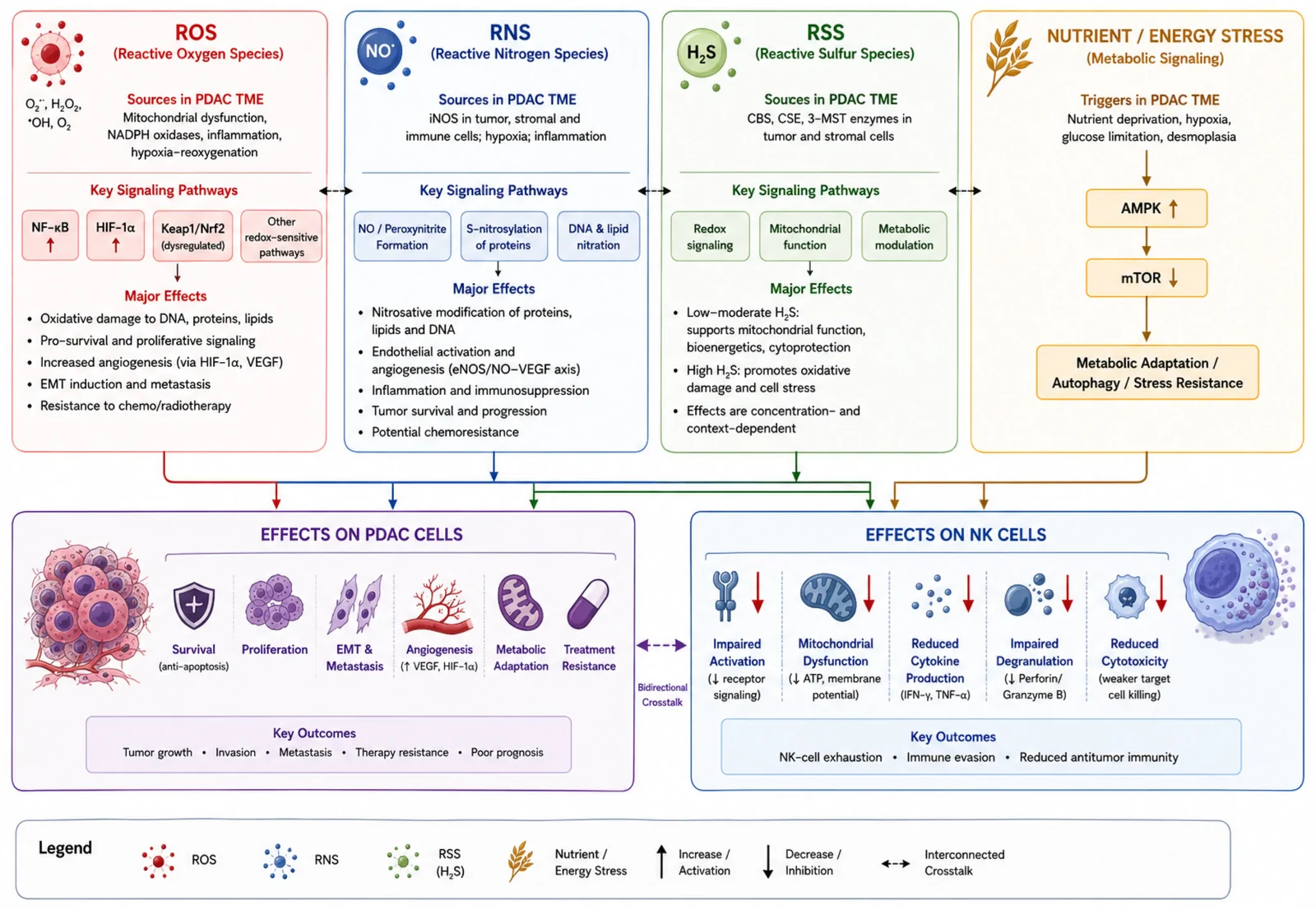
Interaction between reactive species stress, nutrient stress, PDAC metabolism, and NK-cell activity. The figure illustrates the interconnected effects of reactive oxygen species (ROS), reactive nitrogen species (RNS), reactive sulfur species (RSS), nutrient stress, and metabolic signaling on PDAC cells and NK-cell function. ROS-related signaling: Increased ROS in the PDAC tumor microenvironment can activate redox-sensitive pathways, including NF-κB, HIF-1α, and Keap1/Nrf2, contributing to oxidative damage, tumor survival, proliferation, angiogenesis, epithelial–mesenchymal transition (EMT), and treatment resistance. RNS-related signaling: RNS, particularly nitric oxide (NO), contribute to nitrosative signaling through modification of proteins, lipids, and DNA and may promote angiogenesis, inflammation, tumor survival, and treatment resistance. RSS-related signaling: RSS, particularly hydrogen sulfide (H_2_S), participate in redox and metabolic regulation. Their effects are concentration- and context-dependent, with lower levels potentially supporting mitochondrial function and cellular adaptation, whereas excessive H_2_S may promote oxidative damage and cellular stress. Nutrient and metabolic stress: Nutrient deprivation, hypoxia, and metabolic stress can activate AMPK and suppress mTOR signaling, promoting metabolic adaptation, autophagy, and stress resistance in PDAC cells. Altered nutrient availability and persistent redox stress may also impair NK-cell activation, mitochondrial function, cytokine production, degranulation, and cytotoxicity. Overall, the figure highlights the interconnected effects of reactive species and nutrient stress on PDAC progression and NK-cell-mediated antitumor activity.

In PDAC, ROS, RNS, and RSS contribute to tumor progression, immune evasion, metastasis, and therapy resistance through interconnected redox signaling pathways [6,7,8,9]. ROS activate oncogenic pathways involved in proliferation, survival, angiogenesis, and EMT, whereas RNS, particularly NO, influence angiogenesis, inflammatory signaling, and immune modulation [6,7]. RSS, including H_2_S, also participate in redox regulation and metabolic adaptation within the TME and may exert either pro-tumor or antitumor effects depending on their concentration and cellular context [8,10]. These pathways are not independent; rather, ROS, RNS, RSS, and nutrient-sensing pathways interact within the PDAC TME to influence both tumor cell adaptation and NK-cell function. ROS, RNS, and RSS interact with nutrient-sensing pathways and immune-cell metabolism to create a complex network that promotes tumor survival while suppressing NK-cell activity in PDAC. Importantly, ROS, RNS, and RSS should not be considered isolated pathways. These reactive species can interact with one another and with nutrient-sensing pathways, including AMPK and mTOR, thereby linking redox imbalance to metabolic adaptation and immune dysfunction. In the PDAC tumor microenvironment, this interconnected network may simultaneously support tumor-cell survival and suppress NK-cell metabolic fitness and cytotoxic function (Figure 2). ROS are generated as by-products of mitochondrial metabolism and inflammatory signaling, and their production is frequently elevated in PDAC cells due to metabolic reprogramming and hypoxic stress [5]. Increased ROS levels activate signaling pathways such as NF-κB and HIF-1α, promoting tumor cell proliferation, angiogenesis, metastatic potential, and resistance to apoptosis [6]. Although moderate ROS levels may contribute to immune activation under physiological conditions, persistent oxidative stress within the PDAC TME suppresses antitumor immunity and contributes to therapeutic resistance [6].

RNS also play an important role in PDAC biology. Nitric oxide and related nitrogen intermediates can modify proteins, lipids, and DNA through nitrosative reactions, thereby altering signaling pathways involved in tumor survival and immune regulation [7]. NO has been implicated in angiogenesis and metastatic progression through activation of endothelial nitric oxide synthase in tumor-associated vasculature [7]. Furthermore, RNS may contribute to chemoresistance by promoting survival signaling and reducing sensitivity to cytotoxic agents such as gemcitabine [15].

RSS are increasingly recognized as important regulators of redox signaling and cancer metabolism. In PDAC, H_2_S is produced primarily by cystathionine γ-lyase and cystathionine β-synthase [8]. Low concentrations of H_2_S may support tumor growth, mitochondrial function, and cellular bioenergetics, whereas excessive accumulation can induce oxidative damage and cell death [16]. RSS also influence immune regulation within the TME by modulating inflammatory pathways and macrophage polarization [8]. Importantly, ROS, RNS, and RSS interact extensively, forming a complex redox network that shapes tumor behavior, immune suppression, and treatment responses in PDAC [17].

### 3.2. Redox Effects in the PDAC TME

The PDAC TME is characterized by hypoxia, chronic inflammation, metabolic stress, and excessive production of reactive species, all of which contribute to persistent redox imbalance [4,5,6,7,8]. Oxidative stress occurs when the production of reactive species exceeds the cellular antioxidant capacity, resulting in damage to DNA, proteins, lipids, and mitochondrial structures. In contrast, redox signaling refers to the physiological use of reactive species as signaling molecules that regulate cellular adaptation, proliferation, metabolism, and immune responses.

Within PDAC, sustained oxidative stress promotes genomic instability, inflammatory cytokine production, angiogenesis, epithelial–mesenchymal transition, and resistance to chemotherapy and radiotherapy [6,34,35,36]. Activation of redox-sensitive signaling pathways such as NF-κB, HIF-1α, Keap1/Nrf2, and STAT3 further supports tumor survival and adaptation to hostile metabolic conditions [6,13,18,37,38,39]. The hypoxic and nutrient-deprived TME also stimulates metabolic reprogramming, enabling PDAC cells to tolerate oxidative stress and evade apoptosis.

Redox dysregulation significantly affects immune-cell function within the TME. Excessive ROS and RNS impair NK-cell activation, proliferation, cytokine secretion, and cytotoxicity [9,10]. Oxidative stress can also reduce expression of activating receptors such as NKG2D while disrupting perforin and granzyme-mediated tumor killing [13]. Consequently, persistent redox stress contributes not only to PDAC progression but also to immune escape and reduced responsiveness to immunotherapy.

### 3.3. Metabolic Signaling

**Figure 3:**
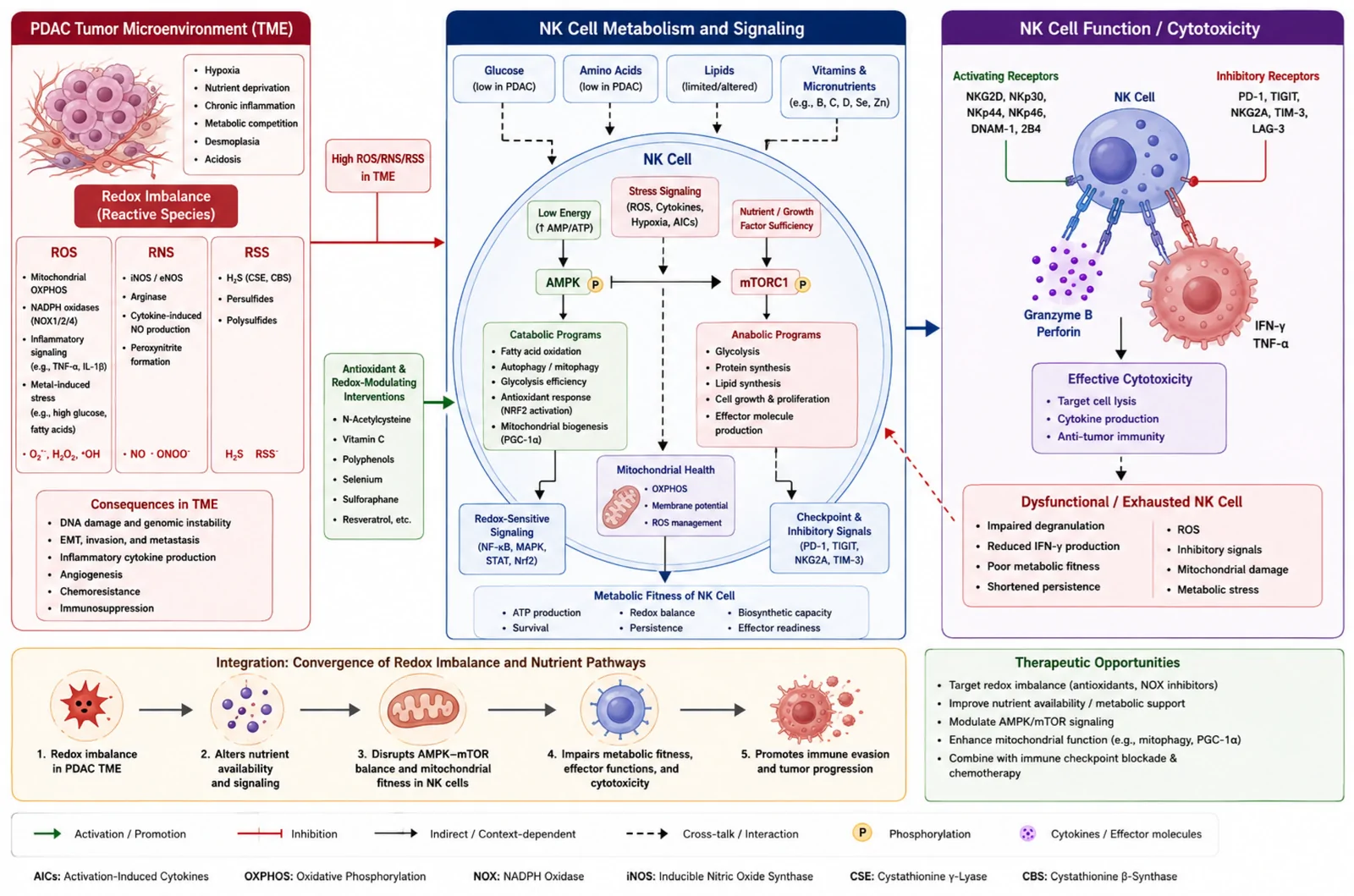
Integration of redox imbalance and nutrient-sensing pathways in regulating NK-cell metabolism and cytotoxicity in PDAC. This schematic illustrates how pancreatic ductal adenocarcinoma (PDAC) shapes natural killer (NK) cell function through coordinated disruption of redox homeostasis and nutrient-sensing signaling pathways within the tumor microenvironment (TME). Elevated levels of reactive oxygen species (ROS), reactive nitrogen species (RNS), and other oxidative stress mediators contribute to redox imbalance, leading to impaired NK-cell survival, metabolic fitness, and cytotoxic activity. In parallel, altered nutrient availability and dysregulated signaling through key pathways such as PI3K–AKT–mTOR, AMPK, and HIF-1α modulate glycolysis, mitochondrial function, and energy metabolism in NK cells. Tumor-derived immunosuppressive factors, including cytokines and metabolic byproducts, further suppress NK-cell activation, degranulation, and cytokine production (e.g., IFN-γ). The combined effects of oxidative stress and nutrient-sensing pathway reprogramming result in NK-cell dysfunction and reduced antitumor immunity in PDAC. This integration highlights potential therapeutic targets aimed at restoring NK-cell metabolic capacity and enhancing cytotoxic responses against tumor cells.

Nutrient sensing pathways play a critical role in both PDAC progression and immune-cell function. Cancer cells within the TME are exposed to hypoxia, nutrient deprivation, and metabolic stress, requiring adaptive mechanisms that promote survival under hostile conditions [18,40]. Among the most important regulators of metabolic adaptation are mTOR and AMPK, which coordinate anabolic and catabolic responses according to cellular nutrient and energy availability. Dysregulation of nutrient-sensing pathways not only supports tumor adaptation but also profoundly influences NK-cell metabolism and effector function through interconnected signaling networks involving PI3K–AKT–mTOR, AMPK, and HIF-1α (Figure 3).

mTOR functions as a central nutrient sensor regulating cell growth, protein synthesis, lipid metabolism, autophagy, and immune responses [11,20]. Persistent activation of the PI3K/AKT/mTOR pathway is common in PDAC and contributes to tumor progression, metabolic adaptation, and treatment resistance [41,42]. Although mTOR inhibitors such as rapamycin, everolimus, and temsirolimus have demonstrated antitumor activity in preclinical studies, clinical responses in PDAC have generally been limited due to compensatory signaling and adaptive resistance mechanisms [12,43].

In NK cells, mTOR signaling is essential for cellular maturation, metabolic fitness, cytokine production, and cytotoxic activity [41,44]. Experimental disruption of mTORC1 or mTORC2 impairs NK-cell development and reduces antitumor function [44]. Excessive metabolic stress within the PDAC TME may therefore compromise NK-cell activity partly through dysregulation of mTOR-dependent signaling pathways.

AMPK acts as a cellular energy sensor activated during metabolic stress and ATP depletion [26]. In contrast to mTOR, AMPK promotes catabolic pathways that restore energy homeostasis while suppressing energy-consuming anabolic processes. Although AMPK was initially regarded primarily as a tumor suppressor, recent evidence indicates that cancer cells may also exploit AMPK signaling to survive under nutrient-limited conditions [27]. In immune cells, AMPK contributes to mitochondrial maintenance, oxidative metabolism, and adaptation to metabolic stress.

Emerging evidence suggests that AMPK signaling may help preserve NK-cell function within the metabolically hostile PDAC TME. AMPK activation has been associated with improved NK-cell survival, metabolic adaptation, cytokine production, and tumor infiltration [20,26,27]. Consequently, modulation of AMPK and mTOR pathways represents a potential strategy to enhance NK-cell-mediated antitumor immunity in PDAC, although clinical validation remains limited.

### 3.4. Mitochondrial Dysfunction and NK-Cell Exhaustion in PDAC

Mitochondrial integrity is essential for NK-cell metabolism, persistence, and cytotoxic function. NK cells require efficient mitochondrial activity to support ATP production, cytokine secretion, and release of cytotoxic granules such as perforin and granzyme B [26,45]. However, the metabolically hostile PDAC TME exposes NK cells to chronic hypoxia, oxidative stress, nutrient deprivation, and lactate accumulation, all of which contribute to mitochondrial dysfunction and metabolic exhaustion.

Persistent exposure to oxidative stress can impair mitochondrial respiration and increase intracellular ROS accumulation in NK cells, leading to reduced proliferation and diminished antitumor activity [46,47]. Metabolic exhaustion also alters NK-cell signaling pathways involved in cytokine production and tumor recognition. Reduced mitochondrial fitness has been associated with impaired IFN-γ secretion, defective cytotoxic granule release, and decreased tumor infiltration [46]. These effects are closely linked to alterations in nutrient-sensing pathways and redox homeostasis that collectively regulate NK-cell metabolism, survival, and cytotoxicity (Figure 3).

In addition to redox stress, competition for glucose, amino acids, and lipids within the TME further compromises NK-cell metabolism [45]. PDAC cells consume large quantities of nutrients through aerobic glycolysis and altered lipid metabolism, creating a nutrient-poor environment that limits NK-cell functionality. Lactate accumulation and acidic pH further suppress NK-cell cytotoxicity and promote immune evasion.

Recent studies suggest that targeting mitochondrial dysfunction and metabolic exhaustion may improve NK-cell persistence and antitumor activity in PDAC [13,46]. Strategies aimed at restoring redox balance, enhancing mitochondrial fitness, or modulating AMPK/mTOR signaling are therefore being investigated as potential adjuncts to NK-cell-based immunotherapy.

## 4. Micronutrient–NK-Cell Interactions in PDAC

Micronutrients, including vitamins and minerals, are essential in small amounts for maintaining proper bodily function. In this section, we explore how micronutrients interact with redox stress and nutrient sensing, and how they influence tumor progression and resistance—specifically through the triple suppression of NK-cell functions: proliferation, cytotoxicity, and tumor infiltration [14]. This discussion also underscores the significant potential for future mechanistic studies to further elucidate these relationships.

**Figure 4:**
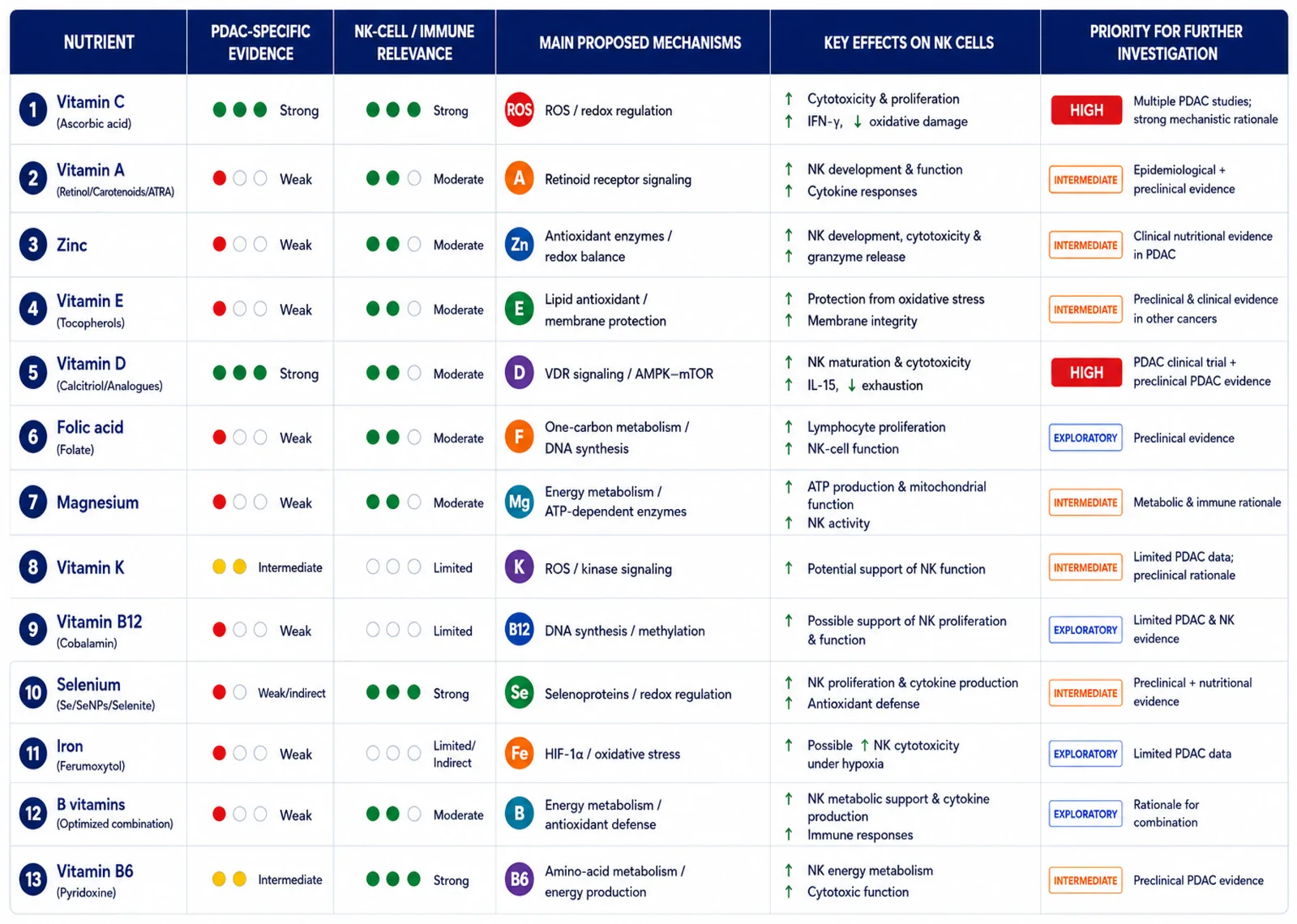
Evidence landscape of nutrient-based interventions relevant to NK-cell function and PDAC. This figure summarizes the nutrients discussed in Table 1 according to the strength of PDAC-specific evidence, relevance to NK-cell function, proposed biological mechanisms, and priority for further investigation. Prioritization reflects the current evidence landscape and mechanistic relevance rather than established clinical efficacy and is intended to guide future research, not to support routine nutrient supplementation in PDAC.

The available evidence is summarized in Table 1 and Figure 4, which separately presents preclinical, clinical, and miscellaneous evidence, respectively.

### 4.1. Vitamins

Vitamins are essential micronutrients that the human body cannot synthesize in adequate quantities and therefore must be obtained through the diet. The fat-soluble vitamins (A, D, E, K) are crucial for many different processes, including immunity. All other vitamins are water-soluble [65,81].

**Figure 5:**
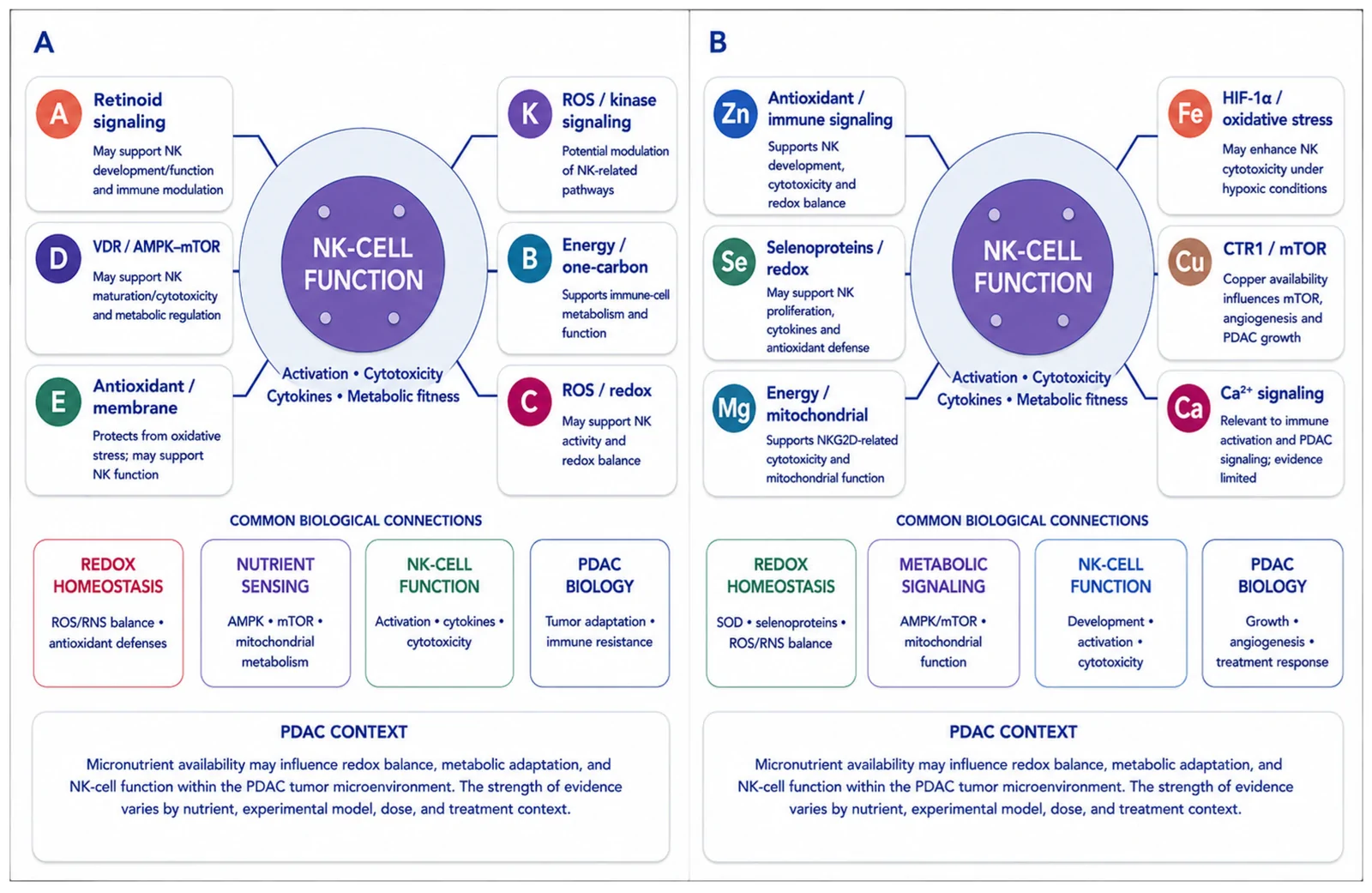
Vitamin/mineral–NK-cell interactions in PDAC. (**A**) Vitamin–NK-cell interactions in PDAC. Schematic overview of the major vitamin-related mechanisms potentially linking micronutrient status with NK-cell function and PDAC biology. Vitamin A, vitamin D, vitamin E, vitamin K, B vitamins, and vitamin C are shown according to their proposed effects on retinoid signaling, VDR/AMPK–mTOR signaling, antioxidant and redox regulation, energy and one-carbon metabolism, and related pathways. These mechanisms may influence NK-cell activation, cytotoxicity, cytokine production, and metabolic fitness, with potential consequences for PDAC tumor adaptation and immune resistance. The figure summarizes mechanistic relationships discussed in the manuscript and does not imply established clinical efficacy of vitamin supplementation in PDAC. (**B**) Mineral–NK-cell Interactions in PDAC. Schematic overview of the major mineral-related mechanisms potentially linking mineral status with NK-cell function and PDAC biology. Zinc, selenium, magnesium, iron, copper, and calcium are shown according to their proposed roles in antioxidant and redox regulation, mitochondrial and energy metabolism, HIF-1α and mTOR signaling, and calcium-dependent immune signaling. These pathways may influence NK-cell development, activation, cytotoxicity, and metabolic function, as well as PDAC growth, angiogenesis, and treatment response. The figure summarizes mechanistic relationships discussed in the manuscript and does not imply established clinical efficacy of mineral supplementation in PDAC.

The major proposed interactions between vitamins, redox regulation, metabolic signaling, and NK-cell function in PDAC are summarized in Figure 5A.

#### 4.1.1. Vitamin A

The term vitamin A encompasses a group of fat-soluble compounds, including retinol, retinal, retinoic acid (RA), and carotenoids [65]. Vitamin A, which is found in foods such as liver, fish oils, and dairy products, plays an important role in cell growth, differentiation, and immune regulation. Its active metabolite, RA, regulates cellular differentiation, apoptosis, and development through retinoic acid receptors (RARs) and retinoid X receptors (RXRs) [81]. Retinoids have therefore attracted considerable attention for their potential anticancer and immunomodulatory effects [82].

Several studies suggest that vitamin A and its derivatives may influence tumor progression and NK-cell function through redox regulation, nutrient sensing, and immune modulation. Vitamin A regulates ROS production in immune cells, including NK cells, helping maintain redox balance and supporting NK-cell cytotoxicity [83]. RA also modulates nitric oxide synthase activity and may influence RNS-related signaling pathways, which are involved in immune activation and tumor surveillance [84]. Given the established roles of hydrogen sulfide and other reactive sulfur species in inflammatory and immune signaling, RA-mediated changes in cellular redox metabolism may potentially intersect with RSS-dependent pathways; however, direct evidence linking RA to H_2_S-producing enzymes such as cystathionine γ-lyase in immune cells remains limited [17,85].

Vitamin A has also been linked to nutrient-sensing pathways involved in tumor progression. A preclinical study demonstrated that all-trans-retinoic acid (ATRA) reduced cancer stem-cell characteristics, including proliferation and migration, particularly when combined with mTOR inhibitors [13]. These findings suggest that vitamin A derivatives may influence both redox signaling and metabolic adaptation in cancer cells.

Several experimental studies support the immunomodulatory effects of vitamin A on NK cells. Oral beta-carotene supplementation has been shown to enhance NK-cell cytotoxicity in human subjects [50], while other studies reported increased frequencies of NK-associated lymphoid cells following beta-carotene supplementation [66,67]. Vitamin A deficiency has been associated with reduced NK-cell activity and lower IFN-γ production, whereas RA enhances NK-cell function and activation [86]. In a breast cancer mouse model, the combination of ATRA and cryo-thermal therapy improved survival and enhanced NK-cell responses through modulation of myeloid-derived suppressor cells [59]. Furthermore, saffron and natural carotenoids have demonstrated antitumor effects in several experimental models [87].

Vitamin A has also been specifically investigated in PDAC. A meta-analysis of 11 studies involving 2705 cases reported that higher dietary vitamin A intake was associated with a reduced risk of PDAC (RR = 0.839, 95% CI: 0.712–0.988) [88]. RA has been shown to regulate PDAC progression through multiple mechanisms, including modulation of intracellular signaling pathways, reduction of tumor-cell adhesion and migration, and suppression of cancer-associated fibroblast activation [81,89]. Preclinical studies suggest that RA may help limit metastasis and tumor progression within the PDAC TME [89]. Clinical studies have shown modest but variable findings. A phase I study combining RA with histone deacetylase inhibitors demonstrated prolonged stable disease in some patients [90], while ATRA has also been repurposed in combination with gemcitabine/nab-paclitaxel chemotherapy in PDAC treatment [91]. However, other clinical studies did not demonstrate substantial improvements in response rates [92].

Despite these promising findings, evidence regarding vitamin A supplementation in cancer remains inconsistent. Although retinoids are being investigated for cancer prevention and therapy [81,93,94], high-dose beta-carotene supplementation has been associated with increased lung cancer risk, particularly in smokers [95]. Large randomized controlled trials, including the Alpha-Tocopherol Beta-Carotene (ATBC) Cancer Prevention Study and the Beta-Carotene and Retinol Efficacy Trial (CARET), reported increased lung cancer incidence and mortality among smokers and asbestos-exposed individuals receiving beta-carotene and retinyl palmitate supplementation [96,97]. These findings emphasize the importance of dose selection, patient stratification, and clinical context when considering vitamin A supplementation.

Overall, vitamin A and its derivatives appear to influence NK-cell activity, redox signaling, and nutrient-sensing pathways involved in PDAC progression. However, much of the current evidence remains preclinical or observational, and randomized PDAC-specific clinical trials are still lacking. Further mechanistic and translational studies are needed to clarify the therapeutic potential, safety, and clinical applicability of vitamin A-based strategies in PDAC.

#### 4.1.2. Vitamin D

Vitamin D is a fat-soluble micronutrient that includes ergocalciferol (D2) and cholecalciferol (D3), both of which are essential for calcium homeostasis, bone metabolism, and immune regulation [76]. Vitamin D is synthesized in the skin through sunlight exposure and is also obtained from dietary sources such as fatty fish, egg yolks, and fortified dairy products. Its active metabolite, calcitriol (1,25-dihydroxyvitamin D; 1,25D), regulates calcium and phosphate balance while also influencing cellular differentiation, proliferation, apoptosis, and immune responses [98]. Observational studies have associated low vitamin D levels with increased cancer risk, while randomized trials suggest that vitamin D supplementation may reduce cancer mortality rather than cancer incidence [99]. In a secondary analysis of a randomized trial involving 25,871 participants, vitamin D3 supplementation reduced the risk of advanced cancer in adults without baseline cancer, particularly in individuals with normal body mass index [100]. These findings suggest that vitamin D may influence tumor progression and treatment resistance rather than cancer initiation alone.

Vitamin D has been increasingly investigated for its effects on NK-cell biology and immune modulation. Several studies indicate that 1,25D can enhance NK-cell cytotoxicity and increase activating receptors such as NKp30, NKp44, and NKG2D, while also promoting the expression of cytotoxic mediators involved in tumor-cell killing [76,101]. Experimental studies further demonstrated that dietary vitamin D supplementation enhanced NK-cell cytotoxicity in normal-weight mice, potentially through modulation of splenic NK-cell populations [102,103]. Vitamin D has also been shown to improve antibody-dependent cellular cytotoxicity (ADCC) mediated by NK cells [76]. However, the immunological effects of vitamin D remain controversial, as other studies reported reduced NK-cell activity, lower CD69 expression, and decreased production of cytokines such as IFN-γ and TNF-α following vitamin D exposure [76,101]. These conflicting findings indicate that vitamin D-mediated immune regulation is likely context-dependent and influenced by factors such as dose, immune status, and TME.

In addition to immune modulation, vitamin D contributes to redox homeostasis and oxidative stress regulation. 1,25D acts as an antioxidant by supporting mitochondrial function and reducing intracellular oxidative stress [104]. Adequate vitamin D levels may help reduce systemic inflammation and oxidative damage to DNA, proteins, and lipids [104]. Vitamin D also modulates inflammatory pathways involving Toll-like receptors (TLRs) and IFN-γ signaling, thereby limiting excessive ROS production and protecting NK cells from oxidative dysfunction [101,105]. Interestingly, Ravid et al. demonstrated that calcitriol enhanced the antitumor effects of immune mediators and anticancer agents by increasing ROS generation selectively within tumor cells [106]. These findings suggest that vitamin D may help maintain a balanced redox state that supports NK-cell function while sensitizing tumor cells to oxidative damage.

Vitamin D may also influence nutrient-sensing pathways associated with cancer metabolism and immune regulation. One study demonstrated that 1,25D regulates the mTOR pathway through DNA Damage-Inducible Transcript 4 (DDIT4/REDD1), an inhibitor of mTORC1, thereby affecting cellular growth and metabolic adaptation [107]. Another study reported that vitamin D3 promoted autophagy and apoptosis in gastric cancer cells through activation of the p53/AMPK/mTOR signaling pathway [108]. Because AMPK and mTOR are major regulators of NK-cell metabolism and tumor adaptation, these findings suggest that vitamin D may indirectly influence NK-cell function through metabolic reprogramming.

Several preclinical and clinical studies have explored the role of vitamin D in PDAC. Vitamin D analogs, including MART-10 and 1-α,25(OH)2D3, have demonstrated antiproliferative and antimetastatic effects through regulation of cell-cycle progression, apoptosis, AMPK activation, and inhibition of PI3K/Akt signaling and EMT [108,109,110]. Preclinical studies suggest that combining vitamin D analogs with AMPK activation may further inhibit PDAC progression [110]. Epidemiological studies also indicate that low vitamin D levels may be associated with increased PDAC risk, although findings remain inconsistent [109]. Clinical studies evaluating vitamin D analogs in PDAC have produced mixed outcomes, with some trials reporting modest improvement in progression-free survival but limited objective responses [111].

Overall, vitamin D appears to influence NK-cell activity, redox balance, and nutrient-sensing pathways relevant to PDAC progression and immune resistance. However, despite encouraging mechanistic and preclinical findings, the clinical efficacy of vitamin D supplementation or analog-based therapies in PDAC remains uncertain. Heterogeneity in study design, patient characteristics, dosing regimens, and treatment duration has contributed to inconsistent clinical outcomes, making it difficult to define the precise therapeutic value of vitamin D in this setting. A better understanding of the contexts and patient populations most likely to benefit may help guide future clinical applications.

#### 4.1.3. Vitamin E

Vitamin E is another fat-soluble vitamin that comprises eight natural forms, including α, β, δ, and γ isoforms of both tocopherols and tocotrienols [112]. α-Tocopherol has long been regarded as the primary form of vitamin E in dietary supplements due to its higher presence in tissues and greater efficacy compared to other forms of vitamin E. However, γ-tocopherol has demonstrated a notable ability to neutralize reactive nitrogen species and inhibit cyclooxygenase, resulting in stronger anti-inflammatory and anticancer effects than α-tocopherol [113].

Vitamin E acts as a potent antioxidant by neutralizing ROS and protecting cellular membranes from oxidative damage [114]. Sandhu et al. showed that dietary vitamin E reduced mutation frequency in tumor cells, suggesting a protective effect against genetic damage and NO-induced stress [115]. In addition to redox regulation, vitamin E succinate (VES) has been shown to activate autophagy in cancer cells through AMPK activation and mTOR inhibition [116].

Studies involving mice and in vitro human NK-cell systems demonstrated that vitamin E can enhance NK-cell activity [117]. In mice, α-tocopherol supplementation significantly increased NK-cell cytotoxicity, while tocotrienols achieved similar effects at lower concentrations [74]. Vitamin E has also been associated with increased IFN-γ production and restoration of NK-cell function under conditions of immune dysfunction [86].

In 1982, alpha-tocopheryl succinate (alpha-TS) was identified as one of the most effective vitamin E forms for inducing cancer cell differentiation, inhibiting proliferation, and promoting apoptosis [118]. Based on existing literature, vitamin E—particularly γ- and δ-tocopherols and tocotrienols—may possess cancer-preventive and anticancer properties [119].

A pilot clinical study in colorectal cancer patients suggested that vitamin E supplementation could enhance NK-cell function and may have potential as an adjuvant in cancer immunotherapy [74]. Experimental studies further demonstrated that tocotrienols suppress NF-κB signaling, induce apoptosis, and inhibit tumor growth through mitochondrial and caspase-dependent pathways [120].

Despite promising mechanistic and preclinical findings, clinical evidence remains inconsistent. While moderate dietary intake may provide protective effects, several randomized controlled trials reported adverse outcomes with high-dose supplementation [121].

Notably, the Selenium and Vitamin E Cancer Prevention Trial (SELECT) demonstrated that high-dose α-tocopherol supplementation increased prostate cancer risk in healthy men without showing overall cancer-preventive benefit [122]. These findings emphasize the importance of avoiding excessive supplementation and support obtaining vitamin E primarily through dietary sources.

Vitamin E, particularly tocotrienol isoforms, demonstrates promising antitumor activity in PDAC through inhibition of NF-κB signaling, induction of apoptosis, regulation of cell-cycle progression, and modulation of Ras–Raf–MEK–ERK signaling pathways [120]. Among the tocotrienols, δ- and γ-tocotrienol showed the strongest inhibitory effects on PDAC cell growth in vitro and in vivo, with δ-tocotrienol also enhancing sensitivity to gemcitabine chemotherapy [123].

Although these findings are encouraging, most available evidence remains preclinical. Clinical studies in PDAC are still limited, and the therapeutic efficacy, optimal dosing, and long-term safety of vitamin E supplementation in PDAC patients remain unclear [81].

#### 4.1.4. Vitamin K (VK)

Vitamin K (VK) is the last fat-soluble vitamin discussed in this review and is essential for blood clotting, bone health, and cardiovascular function. Blackberries, blueberries, kiwi, leafy greens, and cereal grains are rich sources of VK. It exists mainly as VK-1 (phylloquinone), derived from leafy greens, and VK-2 (menaquinone), derived from animal products and fermented foods [124]. Emerging evidence suggests that VK, particularly VK-2, may also exert anticancer effects by regulating apoptosis, oxidative stress, inflammation, and cellular differentiation [125].

VK suppresses cancer cell proliferation through mechanisms involving c-myc and c-fos regulation, modulation of Bcl-2 and p21 pathways, and inhibition of angiogenesis [124,125]. These pathways are also closely linked to NK-cell survival and activation. Cellular senescence contributes to tumor progression through the senescence-associated secretory phenotype (SASP), while NK cells participate in the clearance of senescent and malignant cells. VK may help preserve NK-cell activity and immune surveillance during aging [126].

Textor et al. demonstrated that reduction of c-Myc or N-Myc expression decreases B7-H6 levels on tumor cells, thereby weakening NKp30-mediated NK-cell activation in melanoma, PDAC, and neuroblastoma models [127]. Additionally, genes such as Bcl-2, c-fos, and p21 are important for NK-cell survival, activation, and proliferation, and VK may support these pathways through modulation of intracellular signaling [128,129,130].

VK also influences oxidative stress and metabolic signaling. One study demonstrated that VK-2 induced apoptosis in bladder cancer cells through ROS generation and activation of c-Jun N-terminal kinase (JNK) and p38 MAPK signaling pathways [131,132]. Furthermore, VK-2 has been shown to induce autophagic cell death through AMPK activation during metabolic stress [133].

Preclinical studies suggest that VK may function as a potential adjunctive anticancer therapy through regulation of apoptosis and stress-related signaling pathways [124,125]. VK-1, VK-2, and VK-3 have all demonstrated the ability to induce apoptosis in PDAC cells through caspase-dependent mechanisms involving ROS generation, intracellular calcium signaling, and modulation of Ras–Raf–MEK–ERK and JNK MAPK pathways [53,134].

Combination treatment with VK-1 and sorafenib enhanced apoptosis in PANC-1, PL-5, and MIA PaCa-2 cells by inhibiting phospho-MEK/ERK signaling while activating the JNK/c-Jun/FasL extrinsic apoptotic pathway [134]. Menadione (VK-3) also rapidly activated caspase-3 and PARP cleavage in PDAC cells, with ROS-dependent ERK activation contributing to cancer-cell sensitization [135,136]. Because NK cells also utilize ROS-dependent ERK signaling during cytotoxic granule release, these findings raise the possibility that VK-mediated redox modulation may indirectly influence NK-cell activity.

Despite encouraging mechanistic and preclinical findings, important limitations remain. Excessive ROS generation may impair NK-cell function and contribute to immune suppression, highlighting the complexity of redox modulation in cancer therapy [134,135,136]. Additionally, VK-3 is synthetic, generates substantial oxidative stress, and has been associated with hepatotoxicity; consequently, it is not approved for human use in many countries [135,136].

Clinical evidence evaluating VK supplementation in cancer patients remains extremely limited. Furthermore, PDAC is frequently associated with hypercoagulability and thromboembolic complications, and many patients require anticoagulation therapy. This raises important safety considerations regarding VK supplementation in PDAC patients and underscores the need for clinical caution.

VK demonstrates promising preclinical activity in PDAC through modulation of apoptosis, oxidative stress, and metabolic signaling pathways [53,134,135]. Its ability to regulate ERK, JNK, and AMPK signaling may influence both tumor-cell survival and NK-cell function within the PDAC TME [137]. However, the current evidence is limited almost entirely to preclinical and mechanistic studies.

At present, there are no clinical trials specifically evaluating VK supplementation in PDAC patients [81,134,135]. Therefore, additional translational and clinical studies are needed to determine its therapeutic efficacy, safety, optimal dosing, and the potential risk of thrombosis, particularly in the context of anticoagulant therapy, before VK can be recommended for routine clinical use in PDAC.

#### 4.1.5. Vitamin B

B vitamins are a group of eight water-soluble vitamins essential for energy metabolism, DNA synthesis, neurological function, and maintenance of healthy blood and immune cells [136,138]. This group includes vitamin B1 (thiamin), B2 (riboflavin), B3 (niacin), B5 (pantothenic acid), B6 (pyridoxine), B7 (biotin), B9 (folate), and B12 (cobalamin), which are obtained from a variety of foods including whole grains, meat, dairy products, legumes, eggs, leafy vegetables, nuts, and seeds [136,138].

Recent evidence suggests that B vitamins play important roles in regulating immune-cell metabolism and NK-cell activity [139,140]. Vitamin B12 deficiency has been associated with reduced NK-cell activity in aged rats, whereas B12 supplementation promoted NK-cell expansion and improved cytotoxicity [54,138,140,141]. B3 has been reported to enhance NK-cell recruitment through increased CD62L expression, while B7 deficiency reduced NK-cell cytotoxicity in patients with Crohn’s disease [86]. In contrast, excessive folic acid intake reduced NK-cell cytotoxicity in aged mice, highlighting the importance of balanced vitamin intake [142].

B vitamins also contribute to redox regulation and DNA protection. Folate and vitamin B12 supplementation have been shown to reduce ROS accumulation and DNA damage in experimental cancer models, whereas folate deficiency may promote oxidative stress and tumor progression [141,143,144]. In addition, thiamin and its derivative benfotiamine reduced oxidative stress and NF-κB-driven inflammation in preclinical studies [141,143,144].

Several studies suggest that B vitamins may influence cancer progression through effects on immunity, oxidative stress, and metabolic signaling pathways. A meta-analysis reported that low folate intake combined with MTHFR C677T polymorphisms was associated with increased breast cancer risk [145]. Clinical studies also identified vitamin B12 as an immunomodulator important for maintaining NK-cell cytotoxicity [146].

Ayuk and Abrahamse demonstrated that photodynamic therapy (PDT), which can utilize vitamin B12-related photosensitizers, suppresses cancer growth partly through inhibition of mTOR signaling [147]. Nutrient competition within the PDAC TME may also influence vitamin B metabolism and immune-cell function. Experimental evidence suggests that high pyridoxine demand by PDAC cells impairs NK-cell activity, while vitamin B6 supplementation combined with inhibition of tumor B6 metabolism improved NK-cell function and reduced tumor growth in PDAC models [64].

Two clinical studies evaluated folinic acid (leucovorin), a folate derivative, in PDAC treatment. In gemcitabine-refractory advanced PDAC, addition of oral leucovorin to S-1 chemotherapy improved progression-free survival and disease control rates [148,149,150]. Similarly, a phase II FOLFIRINOX study demonstrated encouraging response rates and survival outcomes, although significant hematologic toxicity was observed [91].

Despite promising findings, the role of B vitamins in cancer remains complex and sometimes contradictory. Both deficiencies and excessive intake may adversely affect immune regulation and tumor biology [143]. While folate is essential for nucleotide synthesis and immune-cell metabolism, folate pathways are also critical for tumor-cell proliferation. Excess unmetabolized folic acid may impair NK-cell activity and potentially support tumor growth under certain conditions [142].

Importantly, leucovorin used in FOLFIRINOX does not function primarily as a nutritional supplement but rather as a pharmacologic modulator that enhances 5-fluorouracil-mediated inhibition of thymidylate synthase. Therefore, its therapeutic role differs substantially from conventional folic acid supplementation.

Emerging evidence suggests that B vitamins may influence PDAC progression through regulation of oxidative stress, NK-cell metabolism, and nutrient-sensing pathways such as AMPK and mTOR [147,148]. Preclinical findings support potential immunometabolic benefits of targeting vitamin B pathways in PDAC, particularly through modulation of NK-cell function and tumor metabolism.

However, clinical evidence remains limited, and most available studies evaluate B-vitamin derivatives as components of chemotherapy regimens rather than as direct immunonutritional interventions [148,149,150]. Further studies are required to clarify optimal dosing, mechanistic effects, safety, and the distinction between nutritional supplementation and pharmacologic modulation in PDAC treatment.

#### 4.1.6. Vitamin C

Vitamin C (ascorbic acid), another member of the water-soluble vitamin group, plays a vital role in collagen synthesis, immune function, iron absorption, and antioxidant protection against free radicals. Unlike most animals, humans cannot produce vitamin C endogenously, making dietary intake essential. It is abundant in fresh fruits and vegetables, particularly citrus fruits like oranges, lemons, and grapefruits, as well as kiwi, strawberries, bell peppers, broccoli, Brussels sprouts, and tomatoes. Regular intake of vitamin C is important to support tissue repair, wound healing, and overall cellular health [151].

Vitamin C plays a significant role in cancer prevention, progression, and treatment, both for its general health benefits and its ability to act as a pro-oxidant at high doses [152]. Ascorbate has been reported to act as an adjuvant, enhancing the antitumor effects of radiotherapy, chemotherapy, immunotherapy, and radio-chemotherapy by generating ROS and inducing ferroptosis [151,152,153].

Vitamin C also enhances NK-cell activity and supports immune function. Ascorbic acid stimulates NK-cell proliferation in culture systems, making it beneficial for NK cell-based therapies [154,155,156]. A preclinical study demonstrated that low-dose vitamin C promotes demethylation of the Killer Immunoglobulin-like Receptor (KIR) promoter and enhances KIR expression, indicative of NK-cell maturation [157]. High doses of vitamin C have been shown to increase perforin and granzyme B secretion by activated NK cells in mouse models [156].

Vitamin C enhances cytokine expression by promoting the activity of Ten-Eleven Translocation (TET) enzymes, which modify DNA methylation and regulate immune-related gene expression, including NK-cell function. This contributes to activation of the IFN-γ/JAK2/STAT1 signaling pathway, increasing tumor-infiltrating lymphocytes and improving immunotherapy responses [154].

Vitamin C also affects oxidative stress and nutrient-sensing pathways. At pharmacologic concentrations, it selectively generates hydrogen peroxide in tumor cells, causing oxidative stress and ATP depletion while sparing normal cells [81,153,158,159,160]. Another study demonstrated that pharmacologic vitamin C suppresses the mTOR pathway through ROS-mediated degradation of Rictor, a key scaffold component of mTORC2, mediated by FBXW7 and HMOX1 signaling, thereby inducing autophagy and inhibiting tumor growth [159].

Since 1980, the influence of vitamin C on NK-cell activity has been investigated [157]. Early studies reported that ascorbic acid inhibited NK-cell activity in a dose- and time-dependent manner, whereas later studies found that oral supplementation increased NK-cell cytotoxicity through protein kinase C activation [86]. More recent studies confirmed its role in NK-cell maturation, enhanced cytotoxicity, and increased NK-cell activity during H1N1 infection when combined with red ginseng [86].

Preclinical studies in PDAC demonstrated that intravenous vitamin C decreases PDAC cell survival, inhibits tumor growth, and enhances the efficacy of chemotherapy [81,153,158,159,160]. Vitamin C also induces autophagy and interferes with glycolysis and DNA repair pathways, contributing to tumor-cell destruction [159].

Clinical trials combining high-dose intravenous vitamin C with chemotherapy agents such as gemcitabine have shown encouraging results, including tumor-size reduction and improved performance status in some patients [158,160,161]. A review of 23 clinical trials found that intravenous vitamin C (≥15 g) improved quality of life and enhanced antitumor responses when combined with chemotherapy or radiotherapy, particularly in advanced PDAC, breast cancer, and ovarian cancer [162].

A randomized trial proposed that adding high-dose intravenous vitamin C to gemcitabine and nab-paclitaxel may improve survival in metastatic PDAC [77]. A phase I/IIa study further demonstrated that intravenous vitamin C was safe, did not interfere with gemcitabine, and may prolong survival with low toxicity [161].

However, despite these promising findings, vitamin C is not considered curative, and larger randomized studies are still needed to confirm optimal dosing, long-term safety, and survival benefits [75,161,162].

Although normalization of vitamin C levels appears to support NK-cell activity, excessive doses may suppress NK-cell function in some settings [35]. Early experimental studies showed inhibitory effects of ascorbic acid on NK-cell cytotoxicity, whereas later studies demonstrated immune-enhancing properties [86]. These conflicting findings suggest that the immunological effects of vitamin C may depend on dose, timing, and redox conditions within the TME.

Several epidemiological and translational studies suggest a protective role of vitamin C in PDAC. A large retrospective cohort study found a lower incidence of PDAC among individuals receiving vitamin C prescriptions compared with those without supplementation [163]. Additionally, a meta-analysis including 17 studies and 4827 PDAC cases reported that higher vitamin C intake was associated with a lower incidence of PDAC (RR = 0.705) [81,158,160,163,164].

Despite promising mechanistic and early clinical findings, evidence supporting vitamin C as a standard adjunctive therapy in PDAC remains limited. Most clinical studies involve small sample sizes, combination regimens, or early-phase trials. Therefore, further large-scale randomized clinical studies are required before vitamin C can be routinely integrated into PDAC management [75,161,162].

### 4.2. Minerals

Minerals are inorganic elements required by the body in small amounts to perform a wide range of physiological functions, including enzyme activity, bone health, nerve signaling, and immune support [165]. Mineral therapy, which involves the use of essential minerals, has shown potential as an adjuvant in cancer therapy due to its effects on immune function, including the activity of NK cells [166].

The major proposed interactions between minerals, redox regulation, metabolic signaling, and NK-cell function in PDAC are summarized in Figure 5B.

#### 4.2.1. Calcium

Calcium is an essential mineral, with 99% stored in bones and teeth, and the remaining 1% circulating in the blood as calcium ions (Ca^2+^). These ions are crucial for muscle function, nerve signaling, and blood clotting. Proper calcium regulation is key for overall health [167].

Emerging research suggests that calcium, particularly its ion form, may play a role in cancer prevention by influencing cellular signaling, apoptosis, and tumor growth regulation. Intracellular Ca^2+^ levels affect both cancer cell growth and death. Researchers developed pH-sensitive sodium hyaluronate-modified calcium peroxide nanoparticles (SH-CaO2 NPs) that induce calcium overload specifically in tumors, effectively killing cancer cells while sparing normal cells and aiding in tumor imaging and control [168]. Thus, calcium overload in this method does not interfere with cancer treatment.

High Ca^2+^ levels are also essential for NK cells to effectively target and kill cancer cells [169]. NK cells deficient in STIM1/ORAI1 activity show severely impaired calcium entry and are unable to kill target cells, highlighting the importance of this signaling complex for NK-cell function [170].

Calcium signaling also intersects with ROS in cancer, forming a complex network that influences tumor progression and survival. Intracellular Ca^2+^ fluxes regulate oxidative metabolism and ROS production, while oxidative stress can alter calcium homeostasis, leading to either tumor-promoting or tumor-suppressive effects depending on the cellular context [171,172,173]. In addition, alterations in calcium signaling have been linked to the mTORC1 pathway and fatty acid synthesis through the calcium–mTOR/SREBP1 axis [174].

Preclinical studies suggest that dysregulated calcium signaling contributes to cancer progression and treatment resistance. Calcium overload strategies using targeted nanoparticles demonstrated selective tumor-cell killing while preserving normal cells [168]. Experimental evidence also supports the importance of calcium signaling for NK-cell cytotoxicity and immune activation [169,170].

Furthermore, calcium-mediated signaling pathways have been implicated in regulating oxidative stress, apoptosis, and tumor metabolism, highlighting calcium as a potential therapeutic target in cancer [171,172,173,174].

Clinical and epidemiological studies suggest that adequate calcium intake may be associated with a reduced cancer risk. A meta-analysis including eight observational studies found an inverse association between total calcium intake and PDAC risk (RR = 0.83, 95% CI: 0.72–0.97), suggesting that higher calcium intake may lower the likelihood of developing PDAC [175].

In addition, a Phase II randomized study evaluated the combination of BAX2398 with 5-fluorouracil and calcium levofolinate in Japanese patients with metastatic PDAC that had progressed after gemcitabine-based therapy (NCT02697058). This trial investigated whether calcium levofolinate could improve therapeutic efficacy when incorporated into combination treatment regimens.

However, despite promising findings, the precise therapeutic role of calcium supplementation in cancer treatment remains unclear, and further clinical studies are required to determine optimal dosing and safety.

Although calcium signaling is essential for immune-cell activation and tumor-cell apoptosis, excessive calcium accumulation can also damage healthy cells and contribute to cellular toxicity [168]. Similarly, calcium-mediated ROS production may either suppress tumors or promote tumor survival depending on the TME and intracellular signaling context [171,172,173]. These findings suggest that calcium homeostasis must be tightly regulated, as both deficiency and overload may have harmful consequences.

Ca^2+^ signaling plays a key role in the growth and spread of PDAC by regulating cell signaling pathways involved in proliferation, invasion, and resistance to therapy [176,177,178]. Altered calcium signaling has been associated with PDAC progression, and targeting calcium-related pathways, including calcium channel signaling, may improve sensitivity to chemotherapy such as gemcitabine [176,177,178].

Although preclinical and observational studies support the relevance of calcium in PDAC biology, direct evidence for calcium supplementation as a therapeutic strategy remains limited. Current clinical evidence is largely observational or based on combination regimens, making it difficult to determine whether calcium itself provides independent anticancer effects. Therefore, further mechanistic and randomized clinical studies are needed before calcium-targeted approaches can be integrated into routine PDAC treatment.

#### 4.2.2. Iron

Iron is another critical element for the body that helps carry oxygen in the blood and supports energy production, immune function, and overall health. It is a key part of hemoglobin, which transports oxygen to the body’s tissues. Iron is found in foods like red meat, poultry, beans, spinach, and fortified cereals. The body stores it in the liver and spleen. Having the right amount of iron is important, as too little can cause anemia, while too much can harm organs [179].

Iron imbalance affects tumor development and treatment responses. Recent research highlights iron metabolism disorders in tumors, their role in ferroptosis and ferritinophagy, and the development of iron-based cancer therapies [180].

Iron can also influence the proliferation and activity of immune cells. In a preclinical study, ferumoxytol, an iron replacement product, was combined with NK cells, inducing ferroptosis in cancer cells and enhancing NK-cell cytotoxicity [180]. Both iron deficiency and iron overload can negatively affect NK-cell function and the overall immune response [181].

Several studies have explored the dual role of iron and ROS in cancer cells. Although iron and ROS are essential for cellular processes, their imbalance can contribute to carcinogenesis. Iron homeostasis strongly regulates ROS production within tumors, and disturbances in this balance may promote tumor progression or treatment resistance [182,183].

Iron also interacts with nutrient-sensing pathways. Iron chelators inhibit mTORC1 signaling by activating the AMPK pathway, with partial involvement of HIF-1/REDD1 and Bnip3 pathways. This effect appears independent of ROS, copper chelation, or PP2A activation, demonstrating that iron availability is necessary for proper mTORC1 activation [184].

Preclinical studies suggest that modulation of iron metabolism may enhance antitumor immunity and reduce cancer progression. Ferumoxytol combined with NK cells promoted ferroptosis and enhanced NK-cell-mediated cytotoxicity against tumor cells [180].

Additional studies demonstrated that iron chelation can inhibit EMT and regulate NDRG1, reducing metastatic potential and treatment resistance in cancer models [185,186,187,188]. Since EMT contributes to tumor migration, immune evasion, and chemoresistance, targeting iron metabolism may represent a potential strategy to improve treatment outcomes.

Iron plays an important role in PDAC, affecting both disease progression and patient health. Patients with PDAC frequently experience anemia and iron deficiency, which may complicate treatment and reduce quality of life [185,186,187,188]. Epidemiological findings regarding iron intake and PDAC risk remain inconsistent, with some studies suggesting that elevated iron levels may increase PDAC risk, whereas others show less clear associations [185,186,187,188].

Although iron modulation and chelation strategies show promising antitumor effects in preclinical and experimental settings, their clinical translation in PDAC remains limited. In patients, evidence is still largely observational, and robust PDAC-specific clinical trials evaluating iron-targeting interventions are lacking. Moreover, the complexity of systemic iron dysregulation in PDAC, including anemia, iron deficiency, and inconsistent associations between iron intake and disease risk, further complicates therapeutic application [185,186,187,188]. Further clinical studies are required to determine the safety, efficacy, and optimal patient selection for iron-modulating strategies in PDAC management [185,186,187,188].

Iron metabolism appears highly relevant in PDAC biology. Ferritinophagy, a process that releases iron from ferritin, has been shown to support PDAC-cell survival and resistance to therapy [185,186,187,188]. Excess iron has also been associated with increased cancer-cell migration, EMT activation, and treatment resistance. Conversely, iron chelation may reduce metastasis and improve treatment sensitivity by regulating EMT-related pathways and NDRG1 expression [185,186,187,188].

Despite promising mechanistic and preclinical findings, direct clinical evidence supporting iron-targeted therapy in PDAC remains insufficient. More translational and randomized clinical studies are required to determine whether iron modulation can safely and effectively improve PDAC outcomes.

#### 4.2.3. Magnesium

Magnesium is an important mineral that helps with energy production, muscle function, and nerve signaling. It also supports healthy bones, blood sugar levels, and a normal heart rhythm. Magnesium can be found in foods like leafy greens, nuts, seeds, whole grains, and legumes. Many people do not get enough magnesium, which can lead to problems like muscle cramps and fatigue. Keeping magnesium levels balanced is important for good health [189].

Magnesium deficiency has been linked to a higher risk of certain cancers. A case-control study indicated that higher magnesium consumption was associated with a reduced risk of breast cancer [190], although understanding its role in cancer development remains complex because of factors such as genotype, nutrient interactions, and inflammation [189]. Hypomagnesemia must be addressed in cancer management, while hypermagnesemia should also be carefully monitored in patients at risk. Therefore, monitoring magnesium levels during supplement therapy is important to avoid adverse effects on cancer treatment.

Magnesium is involved in multiple biochemical reactions and plays a critical role in maintaining immune-system balance. Deficiency in magnesium may reduce NK-cell effectiveness [189]. Research shows that low magnesium levels impair NK-cell activity by affecting the NKG2D receptor, which is essential for NK-cell activation. Studies further demonstrated that magnesium supplementation can restore NK-cell activity and improve immune responses [191].

Magnesium also contributes to redox balance. One study found that higher intracellular magnesium levels protect mitochondria from ROS-induced damage, including hydrogen peroxide-mediated stress [192]. Research on biodegradable magnesium materials further showed reductions in harmful ROS and RNS levels in immune cells, suggesting that magnesium may help control inflammation and protect against oxidative stress [192,193].

In addition to redox regulation, Mg^2+^ has been linked to nutrient-sensing pathways. Mg^2+^ promotes tumor-cell death by activating the AKT/mTOR and Bax signaling pathways, thereby contributing to apoptosis and inhibition of tumor growth [194].

Preclinical studies support the role of magnesium in immune regulation and tumor suppression. Experimental evidence showed that magnesium supplementation restores NK-cell cytotoxicity through regulation of the NKG2D receptor [191]. Other studies demonstrated that intracellular magnesium protects mitochondria from oxidative stress and reduces ROS- and RNS-mediated cellular injury [192,193].

A preclinical study also found that the magnesium transporter protein SLC41A1 was downregulated in PDAC tissues and cells. Increasing SLC41A1 expression in PDAC cells significantly reduced tumor growth in mouse models, suggesting that magnesium transport may represent a potential therapeutic target in PDAC [194].

Several epidemiological studies suggest that adequate magnesium intake may reduce the risk of PDAC. In a cohort study including more than 66,000 men and women aged 50–76 years, every 100-mg decrease in daily magnesium intake was associated with a 24% increase in PDAC risk. Participants consuming less than 75% of the recommended dietary allowance had a 76% higher risk of PDAC compared with those meeting recommended intake levels [195].

Another large prospective study reported that participants in the highest quartile of magnesium intake had a significantly lower risk of PDAC (HR = 0.61; 95% CI: 0.37–1.00; *p*-trend = 0.023) compared with those in the lowest quartile [195]. A separate prospective cohort study among U.S. male health professionals found no overall association between magnesium intake and PDAC risk, although a significant inverse association was observed among overweight individuals (RR = 0.67; 95% CI: 0.46–0.99) [196].

Despite these findings, there are currently no completed clinical trials specifically evaluating magnesium supplementation as a treatment for PDAC. A Phase III clinical trial investigating magnesium sulfate in PDAC patients (NCT01362582) was terminated without conclusive results.

Although magnesium deficiency is associated with impaired immune responses and increased cancer risk, excessive magnesium levels may also be harmful in some clinical settings. Observational studies examining magnesium intake and PDAC risk have shown inconsistent findings, with some studies reporting protective associations while others found no overall relationship [195,196]. These discrepancies may reflect differences in patient populations, metabolic status, dietary factors, and inflammatory conditions.

Magnesium appears to play an important role in PDAC biology through its effects on oxidative stress, NK-cell activation, apoptosis, and mTOR signaling [191,192,193,194]. Experimental studies suggest that magnesium transport pathways, particularly SLC41A1, may suppress PDAC growth [194]. Epidemiological evidence also supports a possible inverse association between magnesium intake and PDAC risk [195,196].

However, direct clinical evidence supporting magnesium supplementation as a therapeutic strategy in PDAC remains limited. Most available data are observational or preclinical, and no definitive clinical recommendations can currently be made. Further mechanistic and randomized clinical studies are needed to determine the safety, optimal dosing, and therapeutic value of magnesium in PDAC management.

#### 4.2.4. Zinc, Copper, and Cadmium

Zinc, copper, and cadmium are biologically important metals whose intracellular balance is tightly regulated by metallothioneins (MTs), cysteine-rich proteins involved in metal transport, storage, and detoxification [197,198]. Zinc is essential for enzyme activity, immune regulation, and DNA synthesis, whereas copper contributes to electron transport, iron metabolism, and antioxidant defense. Cadmium, although toxic, can be partially detoxified through MT binding [197,198].

Elevated MT expression has been reported in several cancers and may contribute to tumor progression, oxidative stress regulation, and chemoresistance [199,200,201,202]. Zinc supports antioxidant enzymes such as superoxide dismutase (SOD), helping neutralize ROS and reduce oxidative damage. Zinc deficiency weakens antioxidant defenses and increases oxidative stress, which may contribute to DNA damage and carcinogenesis [203,204]. Zinc signaling also regulates pathways involved in tumor growth, including MAPK, PI3K/AKT, and mTOR through the zinc transporter ZIP7 [205].

Copper has been linked to tumor progression through activation of mTOR signaling. Increased expression of the copper transporter CTR1 has been observed in PDAC, and copper depletion suppresses mTOR activity, angiogenesis, and tumor growth [206]. In contrast, cadmium exposure promotes inflammation and activates oncogenic pathways such as Notch and AKT/mTOR signaling, contributing to carcinogenesis [207].

Zinc is essential for NK-cell development and cytotoxicity, and zinc deficiency has consistently been associated with reduced NK-cell activity in preclinical studies [208,209,210,211]. Elevated MT expression has also been linked to enhanced NK-cell activity, indicating a role in cancer immune defense [212,213]. However, some compounds that increase MT expression in NK cells may reduce intracellular zinc levels and impair their antitumor function [212].

MT overexpression in tumor cells has additionally been associated with increased NK-cell responses and stronger anticancer immunity [213]. These findings suggest a complex interaction between zinc homeostasis, MT expression, and NK-cell–mediated immune surveillance.

Copper appears to contribute to PDAC progression through activation of mTOR signaling pathways. High expression of the copper transporter CTR1 has been reported in PDAC tissues, and copper depletion suppresses tumor growth and angiogenesis, particularly when combined with mTOR inhibitors such as rapamycin [206].

Studies evaluating MT expression in PDAC have produced conflicting findings. One study demonstrated that low MT1G expression was associated with gemcitabine resistance, while restoration of MT1G reduced stem-like features and improved chemotherapy sensitivity through inhibition of the NF-κB/activin A pathway [214]. In contrast, another study found that high MT expression correlated with aggressive tumor behavior, metastasis, and poorer prognosis in PDAC [213].

Clinical evidence also suggests that zinc deficiency is common in PDAC patients. A 2022 study reported that 17.8% of patients remained zinc deficient after pancreaticoduodenectomy despite receiving zinc supplementation, and lower zinc levels were associated with worse outcomes [215]. Chemotherapy, including gemcitabine-based regimens, has also been shown to significantly reduce zinc levels in PDAC patients, suggesting that monitoring zinc status during treatment may be clinically important [216].

Additionally, a 2024 clinical trial demonstrated that zinc supplementation improved dysgeusia in patients with unresectable PDAC undergoing chemotherapy, potentially supporting nutritional status and quality of life during treatment [217].

Although zinc is required for antioxidant defense and NK-cell function, dysregulated zinc signaling may also support tumor growth through activation of MAPK, PI3K/AKT, and mTOR pathways [205]. Similarly, while MTs may protect against oxidative stress and support immune responses, elevated MT expression has also been associated with chemoresistance and poor prognosis in several cancers [199,200,201,202,213].

Copper depletion strategies show promise in preclinical PDAC models, but copper remains essential for normal cellular metabolism, and excessive depletion may produce systemic toxicity [206]. Cadmium exposure is consistently associated with carcinogenesis and inflammatory signaling, highlighting the importance of minimizing chronic cadmium exposure [207]. Overall, the balance of these metals appears critical, and further clinical studies are needed before targeted metal modulation can be safely integrated into PDAC therapy.

#### 4.2.5. Iodine

Iodine is another trace mineral required for thyroid hormone production, which regulates metabolism, growth, and cellular function. Major dietary sources include iodized salt, seafood, dairy products, and plants grown in iodine-rich soil [218]. Adequate iodine intake is essential for maintaining thyroid function and overall health, while iodine fortification programs have successfully reduced iodine deficiency worldwide [218].

Iodine has antioxidant properties and may contribute to cancer prevention by reducing oxidative damage [219]. However, excessive iodine intake has also been associated with an increased risk of papillary thyroid cancer (PTC), emphasizing the importance of maintaining balanced iodine levels [220]. Iodine toxicity may lead to gastrointestinal, neurological, and thyroid-related complications; therefore, careful monitoring of iodine intake is important in cancer patients [221].

Iodine also influences redox biology. It can protect cells against ROS-mediated damage through antioxidant activity, while under certain conditions it contributes to the formation of reactive iodine species involved in antimicrobial defense and redox signaling [220,221,222]. In addition, excessive iodine exposure may activate the AKT/mTOR pathway and promote PTC progression, whereas moderate iodine levels appear to stimulate proliferation more strongly than very high concentrations [223]. Iodine deficiency has also been shown to regulate thyroid angiogenesis and VEGF expression through mTOR and AMPK signaling pathways [224].

Iodine contributes to innate immune defense against bacterial and viral infections [225]. Several studies suggest that iodine status may influence NK-cell function, although the findings remain inconsistent. Some reports indicate that elevated iodine levels can alter NK-cell activity, whereas radioiodine therapy has been shown to enhance NK-cell–mediated cytotoxicity against tumor cells [226,227,228]. These observations suggest a complex interaction between iodine metabolism and NK-cell immune responses that requires further investigation.

Iodine is currently being explored primarily in PDAC diagnosis and localized treatment approaches rather than as a nutritional therapeutic agent. Imaging studies have shown that iodine uptake measured by CT imaging may help characterize PDAC tumors and monitor treatment responses [229].

In addition, iodine-based radiotherapeutic approaches such as iodine-125 (^125I) seed implantation have been investigated for localized PDAC treatment. These implanted radioactive seeds may help reduce tumor growth and improve pain control through targeted radiation delivery [230]. Experimental approaches using iodine-131 (^131I) incorporated into biogels have also been studied to improve localized radiation targeting while minimizing damage to surrounding healthy tissues [231].

Despite these promising findings, evidence remains limited, and there are currently no large clinical trials demonstrating a definitive therapeutic role for iodine supplementation in PDAC management.

The relationship between iodine and NK-cell activity also remains controversial, with studies reporting both stimulatory and inhibitory immune effects depending on iodine levels and treatment context [226,227,228]. Furthermore, radioiodine-based therapies may provide localized antitumor benefits, but they also carry risks related to radiation toxicity and thyroid dysfunction. Therefore, iodine balance and careful clinical monitoring remain essential when considering iodine-related interventions in cancer patients.

#### 4.2.6. Selenium

Selenium is an essential trace element important for antioxidant defense, immune function, and thyroid hormone metabolism. It is naturally present in foods such as Brazil nuts, seafood, meat, poultry, eggs, grains, and dairy products, although selenium content in plant foods depends largely on soil concentrations [232]. Maintaining adequate selenium levels is important because both selenium deficiency and excess may result in adverse health effects [232].

Selenium has been associated with cancer prevention mainly through its incorporation into selenoproteins, which protect cells from oxidative stress and DNA damage [233]. However, oxidative stress may exert both tumor-promoting and tumor-suppressive effects, and selenoproteins themselves have been implicated in both cancer inhibition and progression [233]. Lobb et al. demonstrated that methylseleninic acid (MSA), an organic selenium compound that acts as a precursor of methylselenol, enhanced the sensitivity of malignant cells to chemotherapy and radiation, although high doses also increased toxicity in normal blood cells [234].

Selenium also regulates redox biology through antioxidant enzymes that neutralize ROS and RNS [235,236]. At physiological levels, selenium supports antioxidant defense and immune function, whereas excessive selenium concentrations may increase ROS production and contribute to cancer cell death [235]. Selenium has additionally been shown to influence key nutrient-sensing pathways. Selenium activates AMPK signaling, suppresses COX-2 expression, and inhibits cancer cell proliferation [237]. Selenium-rich foods and selenium nanoparticles have also demonstrated inhibitory effects on the PI3K/AKT/mTOR pathway in several cancers [238].

Research suggests that selenium supplementation can enhance NK-cell activity and improve tumor cell killing [239,240]. Selenium nanoparticles in particular have shown promising immunomodulatory effects in preclinical studies by enhancing antitumor immune responses [239]. However, both selenium deficiency and selenium excess may impair NK-cell activity, highlighting the importance of maintaining balanced selenium status [240,241].

Experimental studies in mice demonstrated that selenium supplementation significantly increased IL-2 and IFN-γ production, further supporting its role in maintaining balanced immune responses and NK-cell activity [56,57,58,242]. These findings suggest that selenium may contribute to improved innate immune surveillance against tumors.

Selenium has also been investigated in PDAC, particularly as an adjunctive therapeutic strategy. Several studies suggest that selenium compounds may enhance the efficacy of chemotherapeutic agents such as gemcitabine [243]. Selenium deficiency appears to be common among PDAC patients, with some reports indicating low selenium levels in up to 90% of cases [243].

A 2023 study demonstrated that selenium nanoparticles synthesized using *Prunella vulgaris* exhibited antitumor effects against PDAC, supporting the potential of selenium-based nanotherapies [244]. Other studies have reported that selenium may enhance the anticancer effects of juglone and induce ferroptosis-related tumor cell death through selenium-containing compounds such as dibenzyl diselenide (DBDS) [245,246]. However, DBDS acts primarily as a pro-oxidant compound and differs substantially from nutritional selenium supplementation. Therefore, experimental ferroptotic effects observed with DBDS should not be generalized to selenium itself [246].

Despite promising preclinical findings, clinical evidence regarding selenium supplementation in PDAC remains limited and inconsistent. Further studies are needed to determine optimal selenium forms, dosing, safety, and therapeutic efficacy in PDAC patients.

Although selenium is generally associated with antioxidant protection and immune support, excessive selenium exposure may lead to toxicity and increased oxidative stress [232,235]. Selenium compounds may exhibit both antioxidant and pro-oxidant effects depending on concentration, formulation, and cellular context [233,235].

Similarly, while selenium supplementation may enhance NK-cell activity and antitumor immunity, excessive selenium intake can impair immune responses [240]. Current evidence in PDAC remains controversial, with inconsistent epidemiological and experimental findings regarding selenium status and cancer outcomes [56,57,58,243,244,245,246]. Therefore, selenium supplementation should be approached cautiously until larger clinical trials clarify its therapeutic role and safety profile in PDAC.

#### 4.2.7. Phosphorus

Phosphorus is an essential mineral involved in bone formation, energy production, and nucleic acid synthesis. It is obtained from foods such as meat, dairy products, fish, nuts, seeds, and whole grains [247]. Maintaining balanced phosphorus levels is important, as both deficiency and excess may contribute to metabolic and cancer-related complications. Hypophosphatemia is frequently observed in cancer patients, particularly in advanced disease, and may impair tissue oxygenation and immune responses [248]. In contrast, excessive phosphorus levels may suppress vitamin D activity and contribute to tumor initiation [249].

Phosphorus also participates in intracellular signaling, mitochondrial metabolism, and redox regulation. Studies suggest that elevated phosphate levels can increase mitochondrial membrane potential and ROS production, contributing to oxidative stress and vascular dysfunction [250,251]. In addition, cancer cells may adapt to oxidative stress through pathways involving AMPK activation and the pentose phosphate pathway, processes closely linked to phosphate metabolism [250]. mTOR inhibitors commonly used in oncology have also been associated with hypophosphatemia, further highlighting the relationship between phosphorus metabolism and nutrient-sensing pathways [252].

Phosphorus is important for NK-cell development, activation, and migration through phosphorylation-dependent signaling pathways [253,254,255,256,257]. Phosphorus-containing compounds have been shown to enhance NK-cell activity and support immune responses against tumors and infections [253,254,255,256,257]. Low phosphorus levels may impair NK-cell function and potentially weaken immune surveillance against tumor cells [257].

Phosphorus imbalance may influence pancreatic function and PDAC progression. A 2021 preclinical study demonstrated that mice fed a low-phosphate diet developed increased susceptibility to pancreatitis following ethanol exposure due to impaired mitochondrial function and reduced ATP production in pancreatic acinar cells [258]. Postoperative hypophosphatemia has also been reported in patients undergoing pancreatic resection and is associated with increased nicotinamide phosphoribosyltransferase (NAMPT), which promotes renal phosphate wasting [259].

Phosphorus has additionally been explored as a therapeutic modality in PDAC. Phosphorus-32 (^32P) microparticles have been investigated as a targeted intratumoral treatment combined with chemotherapy to slow tumor growth. The OncoSil™ device delivers localized ^32P radiation directly into pancreatic tumors and has shown encouraging preliminary findings [260]. However, further clinical studies are needed to clarify the therapeutic value, safety, and long-term outcomes of phosphorus-based approaches in PDAC.

Although phosphorus is necessary for cellular metabolism and immune function, both deficiency and excess may adversely affect cancer progression and patient health. Hypophosphatemia may impair immune responses and cellular energetics, whereas hyperphosphatemia may contribute to oxidative stress and tumor-promoting pathways [248,249,250,251]. Current evidence linking phosphorus metabolism, NK-cell function, and PDAC remains limited, and additional mechanistic and clinical studies are required before phosphorus modulation can be considered a therapeutic strategy in PDAC.

### 4.3. Prioritization of Nutrients for Further Investigation

Based on the evidence summarized in Table 1, the PDAC-specific evidence for individual nutrients was qualitatively classified as strong, intermediate, or weak. Evidence strength was considered according to the type and consistency of available evidence, degree of PDAC specificity, and level of translational validation. Evidence was considered stronger when supported by direct human PDAC studies and/or substantial direct PDAC evidence with consistent findings and a biologically plausible mechanism. Intermediate evidence reflected direct but limited PDAC preclinical evidence or predominantly preclinical findings with limited translational validation, whereas weak evidence primarily reflected non-PDAC models, indirect associations, or mechanistic evidence without direct PDAC validation. This qualitative classification was used to organize the available literature and does not represent a formally validated evidence-grading system or a meta-analytic quality score.

Research priority was then considered separately from evidence strength. Nutrients were grouped into higher-priority, intermediate-priority, and exploratory categories according to the overall combination of PDAC relevance, immune/redox relevance, mechanistic rationale, and translational potential (Table 2). Thus, a nutrient with limited PDAC-specific evidence could still be considered a priority for further investigation when supported by a strong mechanistic or immune rationale. Priority ranking should not be interpreted as evidence of clinical efficacy or as support for routine supplementation in PDAC.

## 5. Limitations of Current Evidence

Antioxidant modulation in cancer remains biologically complex because ROS may exert both tumor-promoting and tumor-suppressive effects. Several standard chemotherapeutic agents partly rely on ROS-mediated cytotoxicity, raising concerns that indiscriminate antioxidant supplementation could potentially interfere with treatment efficacy in some contexts [261,262,263]. Therefore, the biological effects of micronutrient supplementation may depend on dose, timing, tumor stage, and concurrent therapies.

Another major limitation is the frequent extrapolation of findings from non-pancreatic cancer models to PDAC. The PDAC tumor microenvironment (TME) is characterized by severe desmoplasia, hypoxia, nutrient deprivation, and profound immune suppression, which may substantially alter nutrient availability, redox signaling, and immune-cell responses. Cachexia, malabsorption, systemic inflammation, and metabolic dysregulation further complicate nutrient homeostasis in PDAC patients [4,29].

The evidence is also heterogeneous with respect to nutrient form, dose, disease stage, and treatment context. Different micronutrient isoforms may exert distinct effects on NK-cell function and tumor biology, making direct comparison between studies difficult. Although several vitamins and minerals demonstrate mechanistic and preclinical potential, relatively few studies have demonstrated clinically meaningful benefits specifically in PDAC. Most available evidence remains preclinical, observational, or mechanistic, and definitive clinical evidence supporting routine supplementation in PDAC is currently lacking.

Therefore, antioxidant or micronutrient supplementation should not be viewed as universally beneficial or as a replacement for evidence-based oncologic treatment [46,47,264,265,266]. Carefully designed translational studies and randomized clinical trials are required to determine which nutrients, doses, formulations, and patient populations may provide meaningful benefit.

## 6. Discussion and Future Directions

Recent studies have highlighted the complex role of micronutrients in cancer treatment, particularly through their effects on oxidative stress, metabolic signaling, and NK-cell function. NK cells are important mediators of antitumor immunity, and our previous study shows that NK-cell phenotype and multiple mechanisms of NK-cell suppression play an important role in the immune response within the PDAC TME [14].

The evidence reviewed in this manuscript suggests that micronutrients, redox signaling, and nutrient-sensing pathways should not be considered as independent processes. ROS, RNS, and RSS interact with metabolic pathways such as AMPK and mTOR and may influence NK-cell metabolism, mitochondrial function, cytokine production, and cytotoxic activity. These interactions may simultaneously support tumor-cell adaptation and suppress NK-cell antitumor function.

The use of antioxidants and micronutrients in cancer therapy nevertheless remains controversial because excessive antioxidant supplementation may disrupt physiological redox signaling and potentially interfere with ROS-dependent anticancer effects [46,47,264,265,266]. Therefore, the therapeutic relevance of a given nutrient is likely to depend on the biological context, including nutritional status, nutrient form, dose, disease stage, and concurrent treatment.

A biomarker-guided approach may help address these uncertainties. Measuring micronutrient levels together with redox and oxidative-stress markers and NK-cell signatures could provide a more complete assessment of nutritional, metabolic, and immune status. Because direct measurement of reactive species in patients is challenging, indirect markers such as malondialdehyde (MDA), 8-hydroxy-2′-deoxyguanosine (8-OHdG), and advanced glycation end products (AGEs), together with circulating NK-cell measurements, may provide useful research and monitoring endpoints. Such an approach could help determine whether changes in nutritional status are associated with NK-cell function and treatment response [216].

The nutrients discussed in this review should also be considered according to the strength and relevance of the available evidence rather than as equally supported therapeutic candidates. Table 2 summarizes the proposed prioritization of nutrients for further investigation, while Figure 4 provides a visual overview of the evidence landscape, including PDAC-specific evidence, NK-cell relevance, proposed mechanisms, and research priority. This prioritization is intended to guide future research and does not imply established clinical efficacy.

Future clinical studies should therefore focus on selected nutrients in patients with documented deficiencies or defined biological characteristics rather than applying broad supplementation to unselected PDAC populations. Studies should consider disease stage and biological context and should evaluate different micronutrient isoforms, as they may exert distinct effects on NK-cell function and tumor biology [216]. Further studies are also needed to clarify how micronutrients influence NK-cell proliferation, cytotoxicity, cytokine production, and tumor infiltration in PDAC [14,29].

Particular attention should be given to the interaction between micronutrients, oxidative stress, mitochondrial metabolism, and nutrient-sensing pathways such as AMPK and mTOR [11,14,20,26,27]. These pathways may have opposing effects depending on the cellular context; for example, inhibition of mTORC1 in cancer cells may suppress tumor proliferation and survival, whereas inhibition of mTORC1 in NK cells may impair NK-cell metabolism and cytotoxicity. Understanding these context-dependent effects will be important for developing effective nutrient-based strategies.

The PDAC TME may also limit the delivery and bioavailability of orally administered nutrients. Cachexia, malabsorption, systemic inflammation, and metabolic dysregulation can further affect nutrient availability. Consequently, targeted delivery strategies, including nanoparticle-based vitamin- and mineral-loaded therapies, may warrant investigation, although their clinical application remains experimental [4,29].

Combination strategies integrating selected micronutrient interventions with chemotherapy, immunotherapy, metabolic modulators, or NK-cell-based approaches may also be investigated [13,267]. However, antioxidant supplementation may interfere with ROS-dependent cytotoxic mechanisms induced by anticancer treatments, emphasizing the importance of dose, timing, and patient selection [261,262,263].

A practical future strategy would therefore involve four sequential steps: (1) baseline assessment of micronutrient status; (2) longitudinal monitoring of micronutrients, NK-cell signatures, redox biomarkers, and metabolic markers during standard treatment; (3) prospective testing of selected interventions in biomarker-defined patient groups; and (4) evaluation of treatment response, safety, and immune outcomes. This approach could help move the field from empirical supplementation toward precision nutrition and biomarker-guided intervention.

## 7. Conclusions

The interaction between nutrient availability, reactive species stress, metabolic signaling, and NK-cell function represents a potentially important component of PDAC biology. The evidence reviewed suggests that selected vitamins and minerals may influence redox homeostasis, AMPK/mTOR signaling, mitochondrial function, and NK-cell activity, providing a rationale for further investigation.

However, most current evidence remains preclinical, observational, or mechanistic, and relatively few micronutrients have sufficient clinical evidence to support routine therapeutic application in PDAC.

Therefore, micronutrient supplementation should not currently be considered a substitute for established oncologic treatment.

Future research should prioritize biomarker-guided and controlled approaches integrating nutritional status with NK-cell profiling, redox biomarkers, and metabolic measurements. Such studies should determine which nutritional abnormalities are biologically relevant, which interventions are safe, and which patients are most likely to benefit. Ultimately, carefully designed translational and clinical studies are required to determine whether targeting the redox–nutrient–NK-cell axis can meaningfully complement standard PDAC therapy.

## Figures and Tables

**Table 1:** Summary of Evidence on Nutrient-Based Interventions Relevant to NK-Cell Function and PDAC. The evidence is organized into three sections: (**A**) Preclinical studies, including in vitro, ex vivo, and animal studies; (**B**) Clinical studies, including human observational and interventional studies; and (**C**) Miscellaneous evidence, including computational analyses and systematic or narrative reviews. The table summarizes the nutrient or intervention, proposed molecular mechanisms, effects on NK-cell or immune function, relevance to PDAC, and key limitations of the available evidence. PDAC-specific evidence is qualitatively classified as strong, intermediate, or weak according to the directness and level of PDAC-specific evidence available for each study. Findings from non-PDAC models are included to provide mechanistic context but should not be interpreted as evidence of clinical efficacy in PDAC.

**(A) Preclinical Studies**								
**Year, Reference**	**Study Model**	**Cancer Type**	**Vitamin, Dose**	**Proposed Molecular Target**	**Effect on NK Cells**	**PDAC-Specific Evidence**	**Conclusion**	**Clinical Limitations**
1985, Huwyler et al. [48]	In vitro Human NK cells	Not cancer-specific	Vitamin C (ascorbic acid)	ROS/redox regulation	Dose-dependent modulation of NK-cell activity	Weak	Vitamin C may regulate NK-cell cytotoxicity by modulating intracellular redox balance and ROS involved in target-cell killing.	Human NK-cell study with no cancer-specific or PDAC model.
1986, [Fraker LD et al.] [49]	Wild-type and athymic BALB/c mice	Breast Cancer	Retinol (300–600 µg/day oral administration)	Retinoid/IL-2 signaling	Significant increase in splenic NK-cell activity and cytotoxicity in immunocompetent mice; limited effect in athymic tumor-bearing mice	Weak	Retinol may enhance antitumor immunity by increasing NK-cell activity, contributing to antineoplastic effects.	Breast-cancer model; no PDAC model or human PDAC evidence.
1989 [Prabhala et al.] [50]	In vitro human PMBC	Not cancer-specific	Canthaxanthin (vitamin A carotenoid)	NK activation markers/carotenoid-mediated immune signaling	Increased NK-cell activation markers and enhanced NK cytotoxic activity in cultured PBMCs	Weak	Canthaxanthin may stimulate innate immune responses by enhancing NK-cell activation in vitro.	In vitro human PBMC study with no cancer-specific or PDAC model.
1994 [Ozturk et al.] [51]	N/A	Not cancer-specific	Zinc	Zinc-dependent immune signaling enzymes and transcription factors	Increased NK-cell cytotoxicity after zinc supplementation	Weak	Zinc may enhance innate antitumor immunity by improving NK-cell cytotoxic function.	General immune study with no PDAC model or PDAC cohort.
1997, Colston et al. [52]	Human pancreatic carcinoma cell lines	PDAC	Vitamin D derivatives	VDR/epigenetic signaling	Reduced PDAC cell proliferation and growth; indirect immune relevance	Intermediate	Vitamin D analogs exert antiproliferative effects in pancreatic cancer cells through VDR-mediated signaling pathways.	Direct PDAC cell-line evidence, but limited to an in vitro model with no direct NK-cell assessment.
2010, Showalter et al. [53]	Cell line study	PDAC	Vitamin K (K1 and K2)	Caspase-dependent apoptotic pathways	No direct NK-cell assessment	Intermediate	Naturally occurring vitamin K compounds inhibited pancreatic cancer cell survival through induction of apoptosis.	Direct PDAC cell-line evidence, but limited to an in vitro study with no immune-cell or NK-cell assessment.
2013, [Partearroyo et al.] [54]	Aged rat model	Not cancer-specific	Vitamin B12	One-carbon/mitochondrial metabolism	Decreased vitamin B12 levels were associated with reduced natural killer activity (NKA)	Weak	Adequate vitamin B12 levels may be important for maintaining NK-cell functional activity during aging.	Aged-rat study with no cancer-specific or PDAC model; NK effects were indirectly associated with vitamin B12 levels.
2015, Huijskens et al. [55]	Human NK-cell culture systems	Not cancer-specific	Vitamin C (ascorbic acid)	Redox regulation and NK-cell differentiation pathways	Enhanced NK-cell proliferation and maturation while preserving cytotoxic function	Weak	Vitamin C promotes NK-cell expansion and functional maintenance, supporting potential applications in NK-cell immunotherapy.	Human NK-cell culture study with no PDAC model.
2020, Liu et al. [56]	N/A	Multiple cancer models	Selenium nanoparticles (SeNPs)	Selenoproteins/redox signaling	Enhanced cytotoxic lymphocyte-mediated antitumor responses and immune-cell persistence	Weak	Selenium nanoparticles may enhance antitumor immunity through modulation of redox signaling and immune-cell function.	Multiple-cancer models but no PDAC-specific model; effects were nanoparticle-specific.
2021, Yao et al. [57]	N/A	Immune-cell study	Selenium	GPX4 pathway	Selenium-GPX4 axis protects immune-cell function from oxidative stress	Weak	Selenium-mediated antioxidant pathways may preserve immune-cell viability and function.	Immune-cell study with no PDAC model and no direct NK-cell cytotoxicity assessment.
2021, Razaghi et al. [58]	N/A	Multiple cancer models	Sodium selenite	HLA-E suppression, GPX4-related redox regulation	Enhanced susceptibility of tumor cells to NK-cell-mediated killing	Weak	Selenium may potentiate antitumor immunity by increasing tumor-cell sensitivity to NK-cell cytotoxicity.	Multiple-cancer models with indirect PDAC relevance, but no PDAC-specific validation.
2022, Lou Y et al. [59]	Cell line and mouse model study	Breast Cancer	All-trans retinoic acid (ATRA), 200 µg/day administered from 1 day before to 3 days after cryo-thermal therapy	Retinoic acid receptor (RAR) signaling/NK cytotoxic pathways	Enhanced NK-cell cytotoxicity and improved antitumor immune response after combined ATRA and cryo-thermal therapy	Weak	Vitamin A derivative (ATRA) may enhance NK-cell-mediated antitumor immunity and improve survival when combined with immunogenic therapy.	Breast-cancer cell-line/mouse model with no PDAC validation.
2022, Kim KS et al. [60]	Cell line and animal model study	Prostate Cancer	Ferumoxytol (iron oxide nanoparticle)	IFNG, NKG2D	Increased IFN-γ production and NKG2D expression, enhancing NK-cell cytotoxicity	Weak	Ferumoxytol may potentiate NK-cell-mediated antitumor responses and promote significant tumor regression.	Prostate-cancer model with no PDAC validation; findings may not generalize to dietary iron.
2022, Fu et al. [61]	NK-92 cell culture study	Not cancer-specific	Optimized vitamin B combination	Glucose metabolism, pentose phosphate pathway, cellular bioenergetics	Enhanced NK-cell expansion, viability, and metabolic fitness	Weak	Vitamin B supplementation may support NK-cell growth through metabolic reprogramming and improved energy utilization.	NK-92 cell-culture study with no tumor or PDAC model.
2024, Agura et al. [62]	N/A	Multiple cancer models	Aptamin C/Vitamin C	STAT3, ROS pathways	Enhanced NK-cell anticancer activity and cytotoxicity	Weak	Vitamin C derivatives may enhance NK-cell-mediated antitumor responses.	Multiple-cancer models with no PDAC-specific validation.
2024, He B et al. [63]	Human and murine PDAC models	PDAC		Vitamin D receptor (VDR), DNA methylation, histone acetylation, and Vitamin D-responsive gene networks	No direct NK-cell assessment; enhanced Vitamin D responsiveness in PDAC cells	Intermediate	Epigenetic priming shifted PDAC cells toward a Vitamin D-susceptible phenotype and enhanced the antitumor activity of Vitamin D signaling, suggesting a strategy to overcome therapeutic resistance.	Direct human and murine PDAC models, but primarily preclinical, with no direct NK-cell functional assessment.
2024, [He C et al.] [64]	Cell line study	Pancreatic ductal adenocarcinoma (PDAC)	Vitamin B6 supplement	Glycogen/energy metabolism	Increased NK-cell cytotoxicity and inhibition of tumor growth, breakdown, and energy production	Intermediate	Vitamin B6 may enhance NK-cell antitumor activity and suppress PDAC growth through metabolic reprogramming.	Direct PDAC cell-line evidence with increased NK-cell cytotoxicity, but no in vivo or clinical validation.
**(B) Clinical Studies**								
**Year, Reference**	**Study Model**	**Cancer Type**	**Vitamin, Dose**	**Proposed Molecular Target**	**Effect on NK Cells**	**PDAC-Specific Evidence**	**Conclusion**	**Clinical Limitations**
1991 [Prabhala] [65]	Human supplementation study	Not cancer-specific	β-Carotene (vitamin A precursor)	NK cytotoxicity pathways/β-carotene-mediated immune activation	Increased NK-cell cytotoxicity following β-carotene supplementation	Weak	β-Carotene supplementation may enhance NK-cell functional activity in humans.	Human supplementation study in a non-cancer population; no PDAC cohort or model.
1991 [Watson et al.] [66]	Human supplementation study	Not cancer-specific	β-Carotene (vitamin A precursor)	NK-cell proliferation signaling/β-carotene immune modulation	Increased number of circulating NK cells after β-carotene supplementation	Weak	β-Carotene may enhance innate immune parameters by increasing NK-cell numbers in humans.	Human supplementation study in a non-cancer population; no PDAC cohort or model.
1992 [Garewal et al.] [67]	Human supplementation study	Not cancer-specific	β-Carotene (vitamin A precursor)	β-carotene–associated lymphocyte and NK-cell regulatory pathways	Increased circulating NK-cell number following β-carotene supplementation	Weak	β-Carotene supplementation may support enhancement of innate immune cell populations in humans.	Human supplementation study in a non-cancer population; no PDAC cohort or model.
1996 [Santos et al.] [68]	Human supplementation study	Not cancer-specific	β-Carotene (vitamin A precursor), 50 mg alternate days	β-carotene–mediated immune modulation pathways	Increased natural killer activity in elderly men following long-term β-carotene supplementation	Weak	β-Carotene supplementation may enhance NK-cell functional activity and support innate immune surveillance in humans.	Elderly non-cancer population; no PDAC cohort or model.
1997 [Adachi et al.] [69]	Case report; 16-month-old boy with Shwachman–Diamond syndrome	Not cancer-specific	Vit E	Antioxidant-mediated NK-cell membrane stabilization and signaling	Increased natural killer activity (NKA) following vitamin E supplementation	Weak	Vitamin E supplementation may improve NK-cell function in immunocompromised pediatric patients.	Single-patient case report in a child with a rare genetic disorder; no cancer or PDAC evidence.
1998 [Santos et al.] [70]	Mechanistic study, follow-up analysis	Not cancer-specific	β-Carotene (50 mg alternate days)	Cytokine-mediated NK-cell activation pathways (including IL-12-related signaling)	Enhanced NK-cell activity associated with altered cytokine production and immune-cell communication	Weak	β-Carotene may promote NK-cell activation indirectly through modulation of cytokine networks involved in innate immunity.	Mechanistic follow-up of a non-cancer population; no PDAC cohort or model.
2002, Evans et al. [71]	Phase II trial	Inoperable PDAC	Seocalcitol (EB1089; vitamin D analog)	VDR signaling pathways	Clinical evaluation of vitamin D analog therapy; no direct NK-cell assessment	Strong	Vitamin D analog therapy demonstrated biological activity and acceptable tolerability in patients with advanced PDAC, supporting further investigation of VDR-targeted approaches.	Phase II clinical trial directly conducted in patients with inoperable PDAC.
2006 [Troen et al.] [72]	Human dietary supplementation study	Not cancer-specific	Folic Acid	Folate-dependent methylation and immune-cell proliferation pathways	Folate supplementation increased NKA in women with low-folate diets, but decreased NKA in women with folate-rich diets	Weak	Folate effects on NK-cell activity appear diet-dependent, suggesting a narrow optimal range for immune support.	Human supplementation study in a non-cancer population; no PDAC-specific validation.
2007 [Giacconi et al.] [73]	Patients with atherosclerosis	Not cancer-specific	Intracellular magnesium (Mg) and zinc status	Magnesium- and zinc-dependent immune signaling, cytokine regulation, and NK-cell activation pathways	Reduced intracellular Mg and zinc levels were associated with decreased natural killer activity.	Weak	Adequate intracellular magnesium and zinc levels may be important for maintaining NK-cell function and innate immune competence.	Observational study in patients with atherosclerosis; no PDAC cohort or model.
2007, [Hanson MG et al.] [74]	13 cancer patients	Colorectal cancer (Duke’s stage C and D)	Vitamin E, 750 mg/day for 2 weeks	NKG2D receptor upregulation	Slight but consistent increase in NKG2D expression in all CRC patients following vitamin E supplementation	Weak	Vitamin E may enhance NK-cell activation and could serve as an adjuvant in cancer immunotherapy.	Human cancer study in colorectal cancer, not PDAC.
2012, [Monti et al.] [75]	Phase I clinical trial	Metastatic pancreatic cancer	Intravenous pharmacological ascorbate (vitamin C) combined with gemcitabine and erlotinib	Extracellular H_2_O_2_ generation and redox-mediated selective cancer-cell toxicity	Not directly assessed	Strong	Intravenous pharmacological ascorbate was feasible and well tolerated in combination with standard therapy, supporting further clinical evaluation in metastatic PDAC.	Small Phase I study; limited sample size; no randomized control group; NK-cell activity and immune endpoints were not evaluated.
2023 [christofyllakis et al.] [76]	N/A	Not cancer-specific	Vitamin D	Vitamin D receptor (VDR) signaling and mTOR pathway modulation altered NK-cell gene expression and function	Increased expression of more than 120 NK-cell activity (NKA)-related genes	Weak	Vitamin D may enhance NK-cell-associated transcriptional programs through VDR/mTOR signaling.	General/non-PDAC study examining NK-cell-related gene expression; no PDAC model or cohort.
2024, Bodeker et al. [77]	N/A	Metastatic PDAC	High-dose IV vitamin C, 75 g 3× weekly	ROS, oxidative stress pathways	Improved survival with chemotherapy; possible immune-enhancing oxidative mechanisms	Strong	Pharmacologic ascorbate may improve PDAC outcomes with chemotherapy.	Clinical phase II study directly conducted in patients with metastatic PDAC.
**(C) Miscellaneous Evidence**								
**Year, Reference**	**Study Model**	**Cancer Type**	**Vitamin, Dose**	**Proposed Molecular Target**	**Effect on NK Cells**	**PDAC-Specific Evidence**	**Conclusion**	**Clinical Limitations**
2023, Kietzmann [78]	Review/mechanistic	Multiple cancer types	Vitamin C	TET enzymes, epigenetic regulation, ROS	Supports immune-cell transcriptional regulation and redox balance	Weak	Vitamin C regulates epigenetic and oxidative pathways relevant to NK-cell biology.	Mechanistic review covering multiple cancer types; no direct PDAC nutrient intervention or direct NK-cell experiment.
2024, Ouyang et al. [79]	Bioinformatic/translational	PDAC	N/A	NK-cell marker genes	NK-cell signatures associated with prognosis and immune status in PDAC	Not applicable	NK-cell biology is highly relevant to PDAC progression and prognosis.	PDAC-specific transcriptomic study of NK-cell biology, but not a nutrient intervention; therefore, it should not be used to grade nutrient-specific PDAC evidence.
2025, Mu et al. [80]	Review/mechanistic	Multiple cancer types	Zinc-based nanomaterials (including ZnS)	Glycolysis, mitochondrial metabolism, oxidative stress (ROS), tumor metabolic reprogramming	Indirect immune-metabolic relevance; no direct NK-cell assessment	Weak	Zinc-based nanomaterials disrupt tumor energy metabolism and redox homeostasis, representing a potential strategy for metabolic targeting of cancer.	Indirect PDAC relevance; review-level evidence without direct NK-cell assessment.

**Table 2:** Priority ranking of nutrients for further investigation in PDAC. Nutrients were grouped according to the overall combination of PDAC-specific evidence, immune/redox relevance, mechanistic rationale, and translational potential. Priority categories reflect the current evidence landscape and are intended to guide future research rather than indicate established clinical efficacy or support routine nutrient supplementation in PDAC.

Priority	Nutrients	Why
Higher Priority	Vitamin D, Vitamin C	Direct clinical PDAC evidence together with relevant biological and/or NK-cell evidence supporting further investigation.
Intermediate Priority	Vitamin B6, Vitamin K, Zinc, Selenium, Vitamin E, Magnesium, Vitamin A	Direct but limited PDAC evidence for some nutrients, or substantial NK-cell/immune or redox evidence without direct PDAC validation; further PDAC-specific investigation is warranted.
Exploratory	Folic acid, Vitamin B12, Iron, other B vitamins	Mainly non-PDAC, indirect, or limited mechanistic evidence, with insufficient direct PDAC validation.

## Data Availability

No new data were created or analyzed in this study. Data sharing is not applicable to this article.

## Abbreviations

The following abbreviations are used in this manuscript:

ADCC	Antibody-Dependent Cell-Mediated Cytotoxicity
AGEs	Advanced Glycation End Products
AKT	RAC-alpha serine/threonine-protein kinase
alpha-TS	alpha-TS alpha-tocopheryl succinate
AMPK	AMP-Activated Protein Kinase
ATBC	Alpha-Tocopherol Beta-Carotene
ATP	Adenosine Triphosphate
ATRA	All-Trans Retinoic Acid
Ca.	Calcium
CARET	Beta-Carotene and Retinol Efficacy Trial
CBE	Cystathionine β-synthase
CRC	Colorectal Cancer
CSE	Cystathionine γ-lyase
CT imaging	Computed Tomography Imaging
CTLs	Cytotoxic T lymphocytes
CTR1	Copper Transporter 1 (SLC31A1)
CU	Copper
DDIT4	DNA Damage-Inducible Transcript 4 (also called REDD1)
EMT	Epithelial–Mesenchymal Transition
ERK	Extracellular Signal-Regulated Kinase
FasL	Fas Ligand
FOLFIRINOX	Folinic acid (leucovorin), Fluorouracil (5-FU), Irinotecan, Oxaliplatin
H2S	Hydrogen sulfide
HIF-1α	Hypoxia-inducible factor 1-alpha
IDO	Indoleamine 2,3-dioxygenase
IFNG	Interferon Gamma
IL-2I	Interleukin-2
JNK	c-Jun N-terminal kinase
Keap1	Kelch-like ECH-associated protein 1
KIR	Killer Immunoglobulin-like Receptor
MART-10	Melanoma Antigen Recognized by T cells 10
MDSCs	Myeloid-Derived Suppressor Cells
MEK	Mitogen-Activated Protein Kinase Kinase (MAPKK)
Mg	Magnesium
MHC	Major Histocompatibility Complex
mTORC	Mechanistic Target of Rapamycin Complex
MSA	Methylseleninic acid
MTHFR	Methylenetetrahydrofolate reductase
MTs	Metallothioneins
NADPH	Nicotinamide adenine dinucleotide phosphate (reduced form)
NAMPT	Nicotinamide phosphoribosyltransferase
NDRG1	N-myc downstream-regulated gene 1
NF-κB	Nuclear factor kappa-light-chain-enhancer of activated B cells
NK Cell	Natural Killer cell
NKG2D	Natural killer group 2, member D
NO	Nitric Oxide
Nrf2	Nuclear factor erythroid 2–related factor 2
8-OHdG	8-Hydroxy-2′-Deoxyguanosine
ORAI1	ORAI calcium release-activated calcium modulator 1
OXPHOS	Oxidative Phosphorylation
PARP	Poly(ADP-ribose) polymerase
PBMC	Peripheral Blood Mononuclear Cells
PDAC	Pancreatic Ductal Adenocarcinoma
PD-L1	Programmed Death Ligand-1
PDT	Photodynamic Therapy
PGC-1α	Peroxisome proliferator-activated receptor γ coactivator-1α
PI3K	Phosphoinositide 3-kinase
PTC	Papillary Thyroid Carcinoma
RA	Retinoic Acid
Raf	Rapidly Accelerated Fibrosarcoma kinase
Ras	Rat sarcoma viral oncogene
REDD1	Regulated in Development and DNA Damage Responses 1 (also DDIT4)
RNS	Reactive Nitrogen Species
ROS	Reactive Oxygen Species
RSS	Reactive Sulfur Species
RXRs	Retinoid X Receptors
SASP	Senescence-Associated Secretory Phenotype
SELECT	Selenium and Vitamin E Cancer Prevention Trial
SLC41A1	Solute Carrier Family 41 Member 1
SOD	Superoxide Dismutase
SREBP1	Sterol Regulatory Element-Binding Protein 1
STIM1	Stromal Interaction Molecule 1
STAT3	Signal Transducer and Activator of Transcription 3
TEPA	Tetraethylenepentamine Pentahydrochloride
TET	Ten-Eleven Translocation enzymes
TGF-β	Transforming Growth Factor beta
TIGIT	T-cell Immunoreceptor with Ig and ITIM Domains
TIM-3	T-cell Immunoglobulin and Mucin Domain-containing Protein 3
TME	Tumor Microenvironment
Tregs	Regulatory T cells
VEGF	Vascular Endothelial Growth Factor
VK	Vitamin K
Zn	Zinc
